# A mixed methods approach for measuring topic sensitivity in conservation

**DOI:** 10.1002/pan3.10501

**Published:** 2023-08

**Authors:** Harriet Ibbett, Julia P.G. Jones, Leejiah Dorward, Edward M. Kohi, Asri A. Dwiyahreni, Karlina Prayitno, Stephen Sankeni, Joseph Kaduma, Jesca Mchomvu, Andie Wijaya Saputra, Humairah Sabiladiyni, Jatna Supriatna, Freya A. V. St John

**Affiliations:** 1 School of Natural Sciences, College of Environmental Science and Engineering, Bangor University, Bangor, UK; 2 Tanzania Wildlife Research Institute (TAWIRI), Arusha, Tanzania; 3 Research Centre for Climate Change, Universitas Indonesia, Jakarta, Indonesia; 4 Conservation and Human Behaviour Research Group, Bangor University, Bangor, UK

**Keywords:** bias, free-list, Indonesia, pile-sort, protected areas, psychometric scale, rule-breaking, Tanzania

## Abstract

1. Conservationists increasingly aim to understand human behaviour to inform intervention design. However, obtaining information from people about their behaviour can be challenging, particularly if the research topic is considered sensitive. Topic sensitivity may raise methodological, ethical, political and legal concerns which, if poorly addressed, can have significant impacts on research participants, the research process, data quality and the success of conservation outcomes that are informed by research findings. While considerable effort has been invested in developing techniques for reducing bias when collecting data on sensitive topics, less attention has been focused on identifying if, and why, a topic is sensitive.
2. We use a mixed methods approach to explore how willing people are to discuss topics that could be considered sensitive (e.g. illegal wildlife hunting). Collecting data from people living near protected areas in Indonesia (*n* = 362) and Tanzania (*n* = 345), we developed and tested a psychometric scale to measure topic sensitivity at the respondent level and conducted group exercises (free-lists and pile sorts) to gain a deeper understanding of peoples' willingness to discuss different topics.
3. The perceived sensitivity of topics varied both within and between the two focal contexts, with more topics being perceived as sensitive in Tanzania than Indonesia. Participants' knowledge of rules, and their experiences of living alongside protected areas affected how sensitive they considered topics to be.
4. Mixed methods approaches can provide holistic and nuanced understanding of topic sensitivity. However, recognising that in-depth studies are not always feasible to implement, we demonstrate that methods, such as our Sensitivity Index, can easily be adapted for different contexts and deployed to rapidly obtain valuable insights on topic sensitivity, to help inform conservation research and practice.

## 1 Introduction

Most conservation challenges originate from the actions of people (Balmford et al., 2021). Consequently, conservation science increasingly aims to understand the prevalence and drivers of human behaviours (Cinner, 2018), including those which involve noncompliance with conservation rules (St John et al., 2013). To do so, researchers often use questionnaires and interviews to collect data from people (Bennett et al., 2016), however, obtaining robust information can be challenging, particularly when research topics are sensitive (Tourangeau & Smith, 1996). Participants may refuse to answer, or provide inaccurate responses, resulting in data affected by bias (nonresponse bias and sensitivity bias) (Blair et al., 2020; Tourangeau & Yan, 2007). Asking questions about sensitive topics also has implications beyond data quality, often raising methodological, ethical, political and legal concerns (Lee & Renzetti, 1990), which can impact research participants, the research process, as well as the success of conservation outcomes that are informed by study findings (Brittain et al., 2020). For example, failure to identify and acknowledge topic sensitivity may cause offence, be construed as disrespectful or alienate or endanger those involved (Sieber & Stanley, 1988). Alternatively, researchers may assume some subjects to be more sensitive than they are, resulting in the use of inappropriate or unnecessarily complex methods (Ibbett et al., 2022).

Assessing whether a topic is likely to be sensitive should therefore be an important step when developing conservation research on human behaviour. Yet, while a considerable amount of social science research has addressed the impact of bias when asking sensitive questions (Blair et al., 2020; Krumpal, 2013; Krumpal & Voss, 2020), less attention has been focused on assessing topic sensitivity. Beyond conservation, previous attempts to measure sensitivity have involved asking respondents to rate their ease when discussing different topics (Bradburn et al., 2004), to rate how threatening different topics are (Umesh & Peterson, 1991), and by monitoring factors such as respondents' comfort when answering sensitive questions (Zink et al., 2006).

Various theories exist to describe what makes a topic sensitive (Farquhar & Das, 1999; Krumpal, 2013; Sieber & Stanley, 1988). One of the most widely recognised conceptualisations is that of Lee and Renzetti (1990) who define sensitive research topics as those which present a substantial threat or result in significant costs to those involved, including psychological costs (e.g. feelings of guilt, shame or embarrassment), physical costs (e.g. violence), as well as formal or informal sanctions (e.g. fines or social isolation). Costs may occur because of the content of a response (i.e. admission of a restricted behaviour), but in some situations even the act of participating can be sensitive, regardless of the answer provided.

While any topic has the potential to be sensitive, Lee and Renzetti (1990) argue topics are more likely to be perceived as sensitive if they fall into one of four categories. The first is when research intrudes into private spheres or deeply personal experiences and evokes strong emotional responses; simply asking the question is an invasion of privacy, regardless of the answer (Lee & Renzetti, 1990; Tourangeau & Yan, 2007). In the context of conservation, research about conflict (Redpath et al., 2013), including dispossession of land, violence, law enforcement and the costs of protected areas or living alongside wild-life (Benjaminsen & Bryceson, 2012; Soliku & Schraml, 2018) may stir negative emotions, and force participants to relive traumatic experiences (Thondhlana et al., 2020). Second, a topic may be sensitive if it is concerned with breaking legal or social rules. These topics are sensitive because respondents fear consequences via formal and informal sanctions if they reveal their participation in specific acts (Tourangeau & Yan, 2007). Within conservation, many researchers (e.g. Chang et al., 2019; Fairbrass et al., 2016; Nuno et al., 2013) have investigated illegal wildlife hunting, while others have explored taboos, which govern the harvesting and consumption of wildlife (Alexander et al., 2017; Jones et al., 2008). Third, if the research impinges on the vested interests of powerful elites, it may be sensitive because it presents risks to participants and researchers' safety (Lee & Renzetti, 1990; Robbins, 2000), particularly in contexts of censorship, where media and freedom of speech are restricted. Measuring trends in natural resource use or assessing the effectiveness of conservation policies can produce findings that highlight corruption or abuse of power (e.g. Global Witness, 2015), as well as project success or failure. Finally, sensitivity may arise if the research focuses on topics that are considered sacred (Lee & Renzetti, 1990). For example, conservation has long been interested in documenting local ecological knowledge, however, in some cultures certain knowledge is revered, and participants may fear desecration of long-standing beliefs and traditions, alongside concerns about possible exploitation (Posey, 2002).

Importantly, sensitivity is also defined by the social context in which research occurs (Lee & Renzetti, 1990). What might be an innocuous topic in one context, might be highly sensitive in another. For example, asking questions about illegal behaviour may not be considered sensitive among participants in contexts where conservation laws are poorly enforced and rule-breaking is common practice, but may be highly sensitive in contexts where conservation laws have been imposed or experienced negatively (Razafimanahaka et al., 2012). Social norms, the unwritten rules that prescribe and regulate how people behave, also influence topic sensitivity (Hechtor & Opp, 2001). Norms differ across social classes and subgroups within a society, and their influence on behaviour may vary across cultural orientations (Johnson & van de Vijver, 2002; Lalwani et al., 2006). Actions that deviate from social norms may be perceived by society as unacceptable or undesirable, and result in specific repercussions (e.g. social stigmatisation or ostracism; Ostrom, 1990). In Nigeria, Atuo et al. (2020) found social norms to be a stronger driver of compliance with conservation rules than national legislation. While in Madagascar, cultural prohibitions known as fady dictate different wildlife uses, with studies showing communities are more familiar with fady than national legislation (Keane et al., 2011), meaning research about violating fady may be more sensitive than research on law breaking.

**Figure 1:**
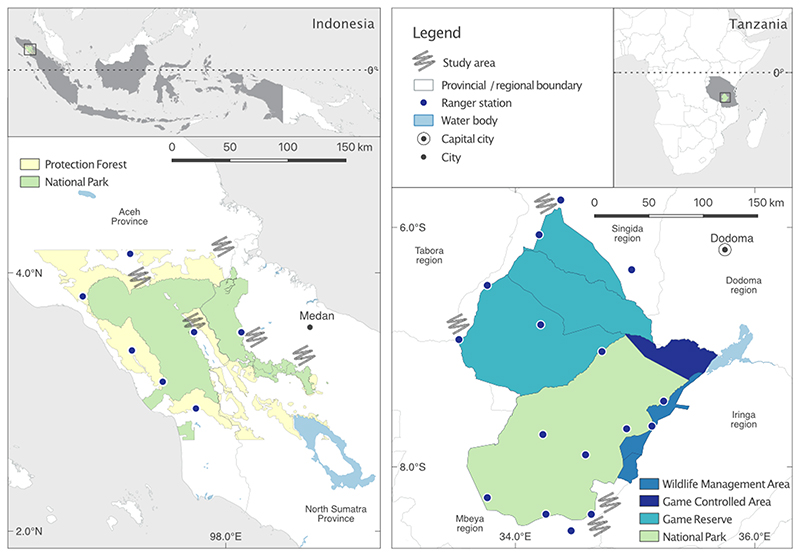
Data were collected around two protected landscapes: the Leuser Ecosystem in northern Sumatra, Indonesia (five locations); and the Ruaha-Rungwa ecosystem in central Tanzania (four locations). In accordance with ethics approval, we do not indicate the precise study locations.

Here, we aimed to explore the sensitivity of different topics relating to natural resource use in two conservation contexts: one in Indonesia, another in Tanzania (Figure 1). Our research was situated within a wider project which focused on understanding drivers of conservation rule-breaking behaviour, using a questionnaire-based study aimed at individuals. Prior to designing the main survey instrument (which is not the subject of this paper), we wished to better understand the context in which the data would be collected, including how willing people living in communities around protected areas would be to discuss natural resource use, including illegal behaviours such as hunting wildlife. Our primary assumption was that any discussions would be regarded as sensitive because of protected area rules limiting natural resource use, and because participants may have preconceptions about our research intentions. We adopted a mixed methods approach to measure how willing people would be to discuss different behaviours in each context, and to explore the usefulness of different methods for assessing overall topic sensitivity.

## 2 Methods

### 2.1 Study sites

Data were collected from five locations (comprised of villages and sub-villages) around the Leuser Ecosystem in northern Sumatra, Indonesia, and four locations around the Ruaha-Rungwa ecosystem in Tanzania (Figure 1). Both landscapes are considered of global importance for biodiversity (Dickman et al., 2014; Myers et al., 2020) and have extensive protected area networks initially established by colonial administrations (Minarchek, 2020; Walsh, 2007). Each landscape encompasses a range of different protected area designations, including community-managed areas (e.g. Wildlife Management Areas in Tanzania), Game Reserves and Game Controlled Areas (Tanzania), Protection Forest (Indonesia) and National Parks (Gunung Leuser National Park, Indonesia and Ruaha National Park, Tanzania). Rules restricting wildlife and natural resource use vary, with the most restricted access to resources in both countries associated with National Parks.

In Indonesia, all wild plant and animal species are classified either as protected or unprotected, with the harvest, capture or destruction of any protected species (regardless of whether it resides in a protected area) prohibited (Article 21, Act No. 5, 1990). Rules regarding natural resource use further depend upon protected area designation and zonation. For example, in the core zone of National Parks any modification of natural habitat is banned (Article 33), while other activities (e.g. tourism or traditional use) are permitted in other zones. In Protection Forests, land clearance is forbidden, and extraction of timber and nontimber forest products is permitted for authorised rights holders or those with licence, and only under certain conditions (Article 50, Law No. 41 on Forestry, 1999).

In Tanzania, all wild animals are property of the state (Article 4, Wildlife Conservation Act No.5, 2009), and it is illegal to hunt, kill or wound any wild animal without permission (Article 55.1). There are strict rules regarding natural resource use in certain protected areas, National Parks can only be entered for the purposes of photographic tourism and Game Reserves allow entrance for photographic tourism and trophy hunting with no other natural resource collection allowed (Wildlife Conservation Act No.5, 2009; National Parks Act, 1975).

### 2.2 Overview of methods

To investigate topic sensitivity, we used a mixed methods approach. Conservation researchers often use questionnaires to ask individuals about sensitive topics (Ibbett & Brittain, 2020), thus it is important to be able to measure topic sensitivity at the individual level. Because sensitivity is a latent construct, meaning it cannot be measured or observed directly (Kyle et al., 2020), we developed and tested a psychometric scale delivered to individuals to assess how sensitive they perceived different topics to be. Furthermore, to improve our understanding of the social context in which the research was occurring, specifically *why* different topics were perceived as sensitive, we conducted group-level exercises. These gathered a diversity of perspectives, plus additional qualitative information with which to triangulate quantitative findings.

### 2.3 Individual psychometric scale measuring topic sensitivity

We conducted a questionnaire with individuals in each study location which gathered basic demographic data (respondent age, gender, years of education) alongside perspectives about the sensitivity of different livelihood activities (Supporting Information, Appendix 1). After reviewing other studies that measured topic sensitivity (Bradburn et al., 2004; Zink et al., 2006) and research on understanding rule-breaking in conservation/environmental protection contexts (e.g. Cialdini, 2007; St John et al., 2015) five items were identified as relevant and formed the basis of our psychometric scale. These items measured injunctive norms (perceptions of how acceptable peers regard the behaviour); the individual’s moral attitude towards the behaviour (whether the individual believes the behaviour is good); whether the behaviour is socially (un)desirable; the individual's own level of (dis)comfort discussing the behaviour; and perceptions of whether community members would be (un)willing to discuss the behaviour. Responses were gathered using 5-point Likert scales (Table 1). Irrespective of the behaviour investigated, we hypothesised that these five items would load onto two factors, one associated with behavioural approval, the other with willingness to discuss the topic.

Respondents were asked about three behaviours in Indonesia (logging inside the National Park, clearing land in the National Park and hunting for wildlife on village land) and four in Tanzania (grazing livestock inside the nearest protected area, eating bushmeat, hunting wildlife on village land and entering the nearest protected area to collect resources), that were identified as present in the landscapes from authors knowledge of the sites, previous literature (e.g. Hariohay et al., 2019) and discussions with protected area managers. In both countries, rules regarding wildlife hunting persist beyond protected areas, therefore we asked about hunting on village land, with a follow up question about how willingness to discuss wildlife hunting might change if conducted in a protected area. We also asked individuals if they knew whether there were any rules associated with each behaviour.

The questionnaire was developed in English and translated into the national languages of Bahasa Indonesia and Kiswahili by two team members fluent in the respective language. An independent back-translation was used to check and revise translation accuracy, with the questionnaire piloted in the field. Questionnaires were administered face-to-face by KP, HS and AWS in Indonesia and SS, JM and JK in Tanzania, and lasted between 10 and 30 min, with respondents given a small, culturally appropriate gift (e.g. phone voucher, or reusable shopping bag) afterwards. Data were collected using Open Data Kit (Brunette et al., 2013) on encrypted mobile phones. We adopted convenience sampling (Newing, 2011), with respondents recruited with the assistance of local guides, based on availability. Wherever possible, the team targeted male respondents aged 18–55, as this was the demographic hypothesised to most likely be involved in hunting, thus information on how willing this group of respondents would be to discuss rule-breaking was of particular interest.

### 2.4 Group exercises

Free-list and pile-sort exercises were conducted in each country. With the help of a local leader, two groups of people (ranging from 6 to 11 participants, with a variety of ages and ethnicities) were convened in each location, with exercises led by one team member, and data recorded by another. Wherever possible, participants differed from those who responded to the questionnaire. Sessions lasted between 1 and 3h (depending on the level of engagement), and participants were reimbursed travel expenses and provided a meal. To encourage active participation, and in recognition of cultural norms, groups were divided by gender, and in Tanzania, separate groups were held for pastoralists and agriculturalists. We considered our framing carefully, emphasising that we were interested in peoples' relationships with protected areas and their rules, rather than whether people broke rules.

#### 2.4.1 Free-lists

Free-lists belong to a suite of methods used to analyse cultural domains, specifically to explore how groups of people think about, and define their world (Puri, 2010). The method is ideal for gathering information about the range and parameters of a specific topic and works by asking respondents to list all the items that come to mind when thinking of a particular topic until the list is exhausted (Guest et al., 2013). Both the item, as described by respondents, and the order the item is listed are recorded. Metrics such as the number of times the item is mentioned across different groups, and the average position in the list (rank) can be used to calculate salience (Guest et al., 2013), a measure which captures the relative importance of an item, with the most salient items those most thought of when the domain is mentioned (Puri, 2010). Free-lists have been successfully used by Harrison et al. (2015) to investigate unauthorised resource use in Ugandan protected areas, and to investigate the cultural salience of different primate species among Waorani people in the Ecuadorian Amazon (Papworth et al., 2013). Two free-list exercises were conducted. In the first, participants were asked to list all the reasons why people from their community went to the protected area. Here we wanted to understand the diverse ways in which local people use protected areas and to explore whether behaviours that breached conservation rules, that we assumed would be sensitive, were openly raised by participants. During the second exercise, participants were asked to list all the challenges faced from living alongside the protected area. Here, our intention was to improve our understanding of the ways in which conservation is perceived.

#### 2.4.2 Pile-sorts

Unconstrained pile-sorts are often used to identify how people classify items and relate them to each other (Guest et al., 2013; Puri, 2010). Drawing on our knowledge of each landscape, and available literature (e.g. Lubis et al., 2020; Walsh, 2007), we generated a list of livelihood activities that we hypothesised may occur adjacent to, or within protected areas in each landscape (Supporting Information, Table S1a). These included activities, such as farming rice or maize, as well as prohibited behaviours, such as killing wildlife or logging for timber. Additionally, we wanted to explore whether specific factors, such as the species killed, the reason for killing wildlife (e.g. for food, income, prestige, livelihood protection) and the technology used (e.g. snare, gun, dog, poison), affected peoples’ willingness to discuss different topics. For each behaviour, we created A4 cards featuring a photograph and a descriptive caption (Supporting Information, Figure S1a). Participants were shown each card in a fixed order and asked as a group to discuss and categorise the behaviour according to how willing they believed people in their community would be to talk about it if the behaviour was conducted on village land. Both the number of piles, and the pile categories were defined by participants. The reason for the allocation into each pile was recorded. Once all cards were allocated to piles according to how willing participants believed people in their community would be to talk about the behaviour depicted, we asked participants how their categorisations might change if the behaviour was conducted in the nearest protected area, noting if any cards moved to other piles. All methods were piloted prior to data collection.

### 2.5 Researcher reflections

In recognition that sensitivity can be influenced by participants' perceptions about who researchers are, throughout data collection, we kept notes reflecting on how participants reacted to our presence. Observations included questions participants asked, comments relating to the research aims, as well as participants' body language and reactions during data collection. To ensure consistency within and between teams, formal training was provided to authors collecting data. Moreover, debriefs were held after each group exercise session.

### 2.6 Ethical considerations

All data collection was anonymous with no personal identifiers collected. To maintain anonymity, free, prior and informed consent was sought from participants verbally, and all participants were aged 18 years or over. Research was approved by Bangor CoESE Ethics Committee (coese2019hi01), and all relevant permissions were granted at national, regional and local levels. Data were collected in Tanzania between September and December 2019, and in Indonesia between August and November 2020. We returned to villages after data collection to share findings from this research, and the wider project, with participants. Rigorous measures were implemented to minimise transmission of COVID-19, with local and national regulations adhered to (Supporting Information, Table S1b,c).

### 2.7 Analysis

#### 2.7.1 Explanatory factor analyses for psychometric scale development

Explanatory factor analysis was conducted following the guidance of Watkins (2018). All rows with missing data were excluded from analysis. Using ‘psych’ (Revelle, 2021) in R (v. 4.0.3; R Core Team, 2021) we created correlation matrixes of the five items constituting our proposed psychometric scale of topic sensitivity and confirmed factorability using Bartlett’s test of sphericity (Bartlett, 1951) and the Kaiser-Meyer-Olkin test (Kaiser, 1974). Parallel analysis (Horn, 1965), and the visual scree test (Cattell, 1966) were used to determine the appropriate number of factors to retain. In both countries, results suggested the possibility of one- or two-factor dimensionality, we thus ran analyses for both options and compared chi-square test of exact fit, root mean square error of approximation (where a RMSEA ≤ 0.06 indicated strong model fit), Tucker-Lewis index (TLI ≥ 0.95 indicated strong model fit), standardised root mean square residual (SRMR ≤ 0.08 indicated strong model fit) and the Bayesian information criteria (BIC) to determine the best model (Boateng et al., 2018). Criteria for determining factor adequacy were established a priori, with factor loadings above 0.40 considered reasonably strong, and loadings of 0.70 or 0.80 very strong (Furr, 2011). Due to the nature of the constructs, we assumed factors would be correlated, therefore, an oblimin rotation was employed (Furr, 2011). To test internal consistency, we calculated raw coefficient alpha and Omega total, with 0.7 considered a reasonable threshold for psychometric scale development (Streiner, 2003).

#### 2.7.2 Sensitivity Index

Using the outcome of the exploratory factor analysis, a Sensitivity Index (i.e. a value from 0 to 1, which indicated how sensitive a topic was) was calculated for each respondent, for each behaviour. Weighted factor scores, that considered correlation between factors, were extracted (Revelle, 2021), and to improve interpretability, were transformed from *z*-scores to a scale between 0 and 1. The ratio of variance represented by each factor was calculated by dividing the proportion of variance described by each factor, by the total variance. The transformed weighted factor scores were then multiplied by the ratio of variance and summed together to create a composite index of sensitivity for each respondent, for each behaviour. The higher the Sensitivity Index, the more sensitive the topic was perceived to be.

#### 2.7.3 Beta regression models

We first summarised the demographics of the sample in each country using descriptive statistics. To examine which variables influenced a respondent's perception of topic sensitivity, we fitted beta regression models with mixed effects (Douma & Weedon, 2019) with a logit-link structure to each country dataset using ‘glmmTMB’ (Brooks et al., 2017). Beta regression models were deemed most suitable for analysing continuous data ranging between 0 and 1 (Douma & Weedon, 2019). The Sensitivity Index was the response variable, with respondent gender, age, years of education, the behaviour, whether the respondent had knowledge of any conservation rules pertaining to the behaviour, and the type of protected area they lived nearest to, included as predictors. We included interactions for each behaviour and the respondents’ knowledge of rules; and each behaviour and the type of protected area the respondent lived closest to. To improve the interpretability of coefficients, continuous variables for respondent age and years of education were scaled and centred by subtracting the mean and dividing by two standard deviations (Gelman & Hill, 2007). The grouping structure of the data, whereby each respondent answered questions about several behaviours, was reflected in the model by including individual respondents as a random effect.

#### 2.7.4 Group exercises

For each of the items listed during the free-list exercises we calculated a Smith’s salience score using ‘AnthroTools’ in R (Purzycki & Jamieson- Lane, 2017; Supporting Information, Appendix 2). For the pile-sort data, the number of piles identified by each group, and the frequency that each card was grouped into a pile across all groups was summarised, with the behaviours ordered and plotted by sensitivity. Although groups were divided by gender (and ethnicity in Tanzania), data were pooled, as we were primarily interested in understanding the range of activities that emerged. Qualitative notes (including researchers’ reflections) made during group exercises were used to triangulate findings and place the results in context.3

## 3 Results

### 3.1 Psychometric scale development

Data for the psychometric scale were collected from 590 people, 302 in Indonesia and 288 in Tanzania. In line with the sampling strategy, the gender of both samples was biased towards men (Indonesia, 75% male, Tanzania, 57%). The median respondent age was 38 years (IQR: 30-48) in Indonesia, and 38 years (IQR: 28-46) in Tanzania. Respondents reported a mean of 9.9 (SE: 0.21) years education in Indonesia, and 6.6 (SE: 0.17) years in Tanzania.

Analysis of the psychometric scale was highly promising. Bartlett's test of sphericity indicated that the correlation matrices were nonrandom (Indonesia: *X*^2^ = 1264.4, *p*<0.001, Tanzania: *X*^2^ = 979.31, *p*<0.001), and the KMO statistics were well above the 0.5 minimum standard for conducting a factor analysis (Indonesia: 0.81, Tanzania: 0.69). In both countries, the two-factor model performed best, with a stronger model fit in Indonesia than Tanzania (Table 2). In both countries, and in line with our hypothesis, three items (injunctive norm, moral attitude and social desirability) loaded onto Factor1, while two items (personal comfort and willingness of community to discuss behaviour) loaded onto Factor2 (Table 2). Measures of internal consistency for Factor1 were reasonable for psychometric scale development in both countries, but just under the ideal threshold for Factor2 in Indonesia, and considerably so in Tanzania. Descriptive statistics and distribution of item responses are shown in Supporting Information, Appendix 2.

**Figure 2:**
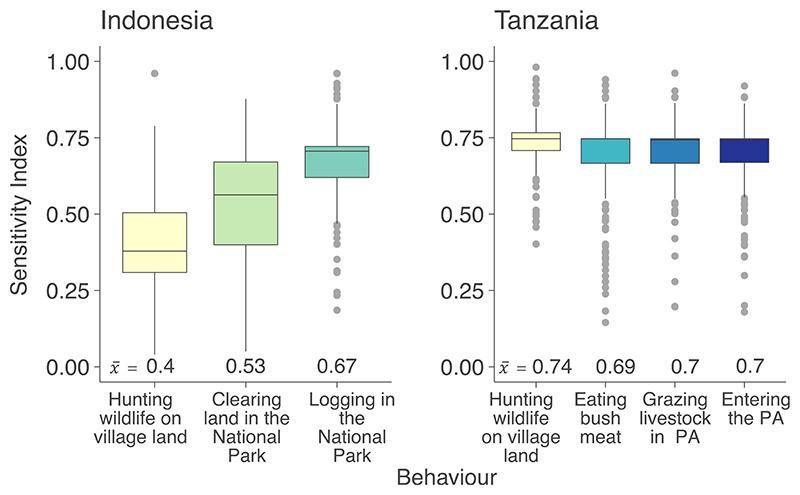
Sensitivity Index for each behaviour assessed in Indonesia (left, *N* = 302) and Tanzania (right, *N* = 288). Different behaviours are represented by unique colours. Thick line indicates the mean score (numeric value at bottom), shaded areas and circles show the distribution of the data. PA = protected area. Scores range from 0 (implying no sensitivity) to 1 (implying high sensitivity).

The Sensitivity Index, created by summing weighted, transformed factor scores derived from our exploratory factor analysis identified that, logging in the National Park was the most sensitive behaviour investigated in Indonesia (mean Sensitivity Index=0.67, [95% CI:0.01]; Figure 2), implying it was a reasonably sensitive topic to discuss; nearly all respondents (97%) were aware of rules prohibiting this behaviour. Clearing land in the National Park obtained a slightly lower mean Sensitivity Index of 0.53 [0.02], suggesting it was less sensitive to discuss; slightly fewer respondents were aware of rules (91%). Hunting wildlife on village land 0.40 [0.01] obtained the lowest Sensitivity Index (Figure 2), with only 65% of respondents reporting knowledge of rules associated with this behaviour. When asked how sensitivity might change when discussing hunting in protected areas, most respondents reported sensitivity would increase a little (62% of respondents) or a lot (10%).

In Tanzania, there was little difference in mean sensitivity indices between behaviours; hunting wildlife on village land obtained the highest Sensitivity Index 0.74 [0.01], closely followed by entering the nearest protected area 0.70 [0.01], grazing livestock in the nearest protected area 0.70 [0.01] and eating bushmeat 0.69 [0.01] (Figure 2). Some respondents reported the sensitivity of discussing hunting, when conducted in the protected area (compared to village land), would increase a little (10%), or a lot (20%), but most (49%) reported sensitivity would stay the same. Overall, respondents in Tanzania reported high awareness of rules, regardless of behaviour (88% of respondents knew of rules about hunting on village land, 91% for eating bushmeat, 95% for grazing livestock, 93% for entering PA).

Modelling showed that in Indonesia, clearing land and logging for timber in the National Park were considered significantly more sensitive than hunting on village land (Table 3). In Tanzania, there was no significant difference in sensitivity between hunting on village land, eating bushmeat and grazing livestock. However, entering the protected area without a permit was considered significantly less sensitive than hunting on village land (Table 3). Those with greater awareness of rules reported behaviours as more sensitive in Indonesia, but not in Tanzania, probably because rules were widely known for all behaviours in Tanzania, meaning there was less variability in the data. Gender was a significant predictor of sensitivity in Indonesia with women more likely to report topics as sensitive than men, but not in Tanzania. Other demographic characteristics including education, and age, along with the type of protected area the respondent lived nearest to, were not significant predictors of sensitivity in either country. There were no significant interactions in Indonesia, however, in Tanzania those living near a Game Reserve considered entering the reserve without a permit significantly more sensitive compared to those living near the National Park.

### 3.2 Free-lists

#### 3.2.1 Were illegal behaviours freely listed as reasons why people go to protected areas?

In both countries, participants reported entering protected areas for various livelihood supporting activities including to collect firewood, plant materials (e.g. agar, rattan, bamboo, wild cinnamon, wild fruits) or tap trees (rubber, palm) in Indonesia, and to fish, collect firewood, honey, water and building materials in Tanzania (Supporting Information, Table S2g). Overall, the mean number of items listed was lower in Indonesia (5.9 items) than Tanzania (8.1 items). A number of these freely listed activities are prohibited demonstrating that participants were willing to raise these topics with researchers in group settings. In both countries, no item achieved a salience higher than 0.63 (Table S2g). This likely reflects heterogeneity in types of activities conducted across these large landscapes (>7000 km^2^). In Indonesia, the most salient reason reported for going to a nearby PA was to farm, while in Tanzania grazing livestock, collecting timber and fishing were most salient. Groups in both countries reported wildlife hunting as a reason for going to protected areas, although this was not particularly salient in either. In Indonesia, hunting wildlife was referred to in several ways, both broader taxonomic groups (primates) and specific species (wild boar, *Sus scrofa*) were mentioned alongside ‘hunting wildlife’.

#### 3.2.2 What challenges do people face living alongside protected areas?

Overall, participants in Indonesia reported far fewer challenges (10 items, mean 1.4 challenges listed per group) from living alongside protected areas than in Tanzania (25 items, mean 5.1 challenges listed per group), with three groups in Indonesia listing no challenges at all, suggesting that relationships between communities and protected areas were more challenging in Tanzania than Indonesia. In Indonesia, an inability to expand farmland due to the presence of the National Park was most salient, however, overall salience was low (0.3) with the item only mentioned by 3 of 10 groups (Table 4). In Tanzania, challenges associated with living alongside wildlife were the most salient items, with wildlife damaging crops mentioned prominently by nearly all groups (salience 0.69, seven of eight groups; Table 4).

In Tanzania, free-lists revealed differences in the types of challenges experienced across the landscape. For example, around Game Reserves, most of the challenges reported related to the costs of living alongside wildlife (e.g. crop damage, livestock loss, injury and human fatalities; Table 4). Discussions here often became sensitive because they involved respondents recalling traumatic events (e.g. deaths caused by wildlife) or describing emotions, such as fear or anxiety, experienced as a result of living alongside wild-life (Supporting Information). In contrast, groups adjacent to the National Park listed issues such as boundary disputes and discontent at the way rules were enforced with more prominence. Interestingly, two groups here highlighted that threatening to report others to rangers for rule-breaking (e.g. for hunting wildlife) was a particular challenge, suggesting that any discussions about conservation laws or the National Park in these communities were likely to be sensitive, because of communities’ poor perceptions of, and relationships with,National Park authorities, as well as concerns about the repercussions of discussing rule-breaking.

### 3.3 Pile-sorts

#### 3.3.1 How willing were people to discuss different behaviours in Indonesia?

**Figure 3:**
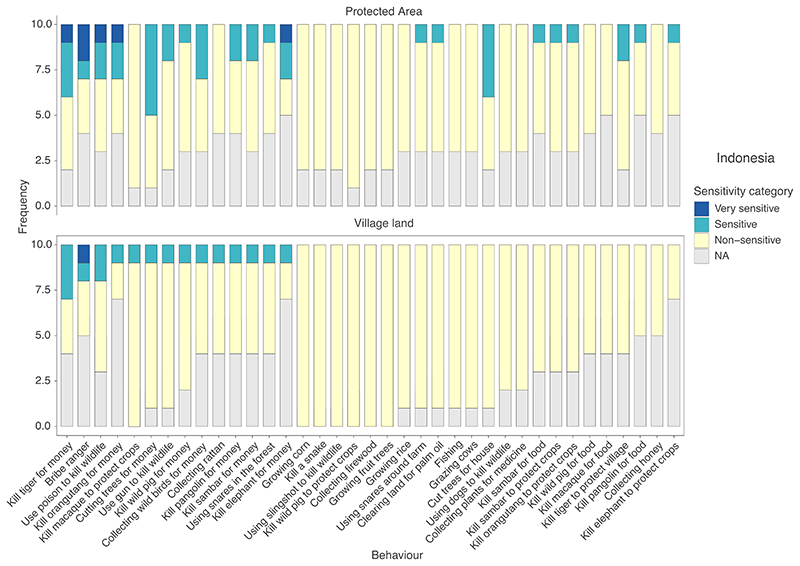
The reported sensitivity of behaviours when conducted in village/community land (bottom) and in protected areas (top) based on pile-sorts conducted by 10 groups with 60 participants living in the Leuser Ecosystem in Indonesia. NA represents behaviours that participants reported they were unaware of, and thus were not able to classify.

Participants in the 10 groups (60 participants) organised the 37 behaviours featured on the pile-sort cards into up to four self-defined categories of sensitivity. These were: very sensitive (participants felt that community members would not discuss the topic openly or honestly); sensitive (community members would be hesitant to discuss the topic); nonsensitive (the behaviour was widely engaged in and community members were willing to talk about it); and not applicable (NA, participants were unaware of the behaviour and thus were unable to comment; Figure 3).

Overall, few behaviours were categorised as very sensitive or sensitive (Figure 3). Most behaviours were categorised as nonsensitive, despite some being prohibited. In four groups, when asked how sensitivity would change if behaviours were conducted in the nearest protected area, participants reported there would be no change in categorisation for any behaviours. In the six other groups, participants reported that more behaviours would become sensitive to talk about, and that the sensitivity of topics that were already sensitive would increase (Supporting Information).

#### 3.3.2 How willing were people to discuss different behaviours in Tanzania?

**Figure 4:**
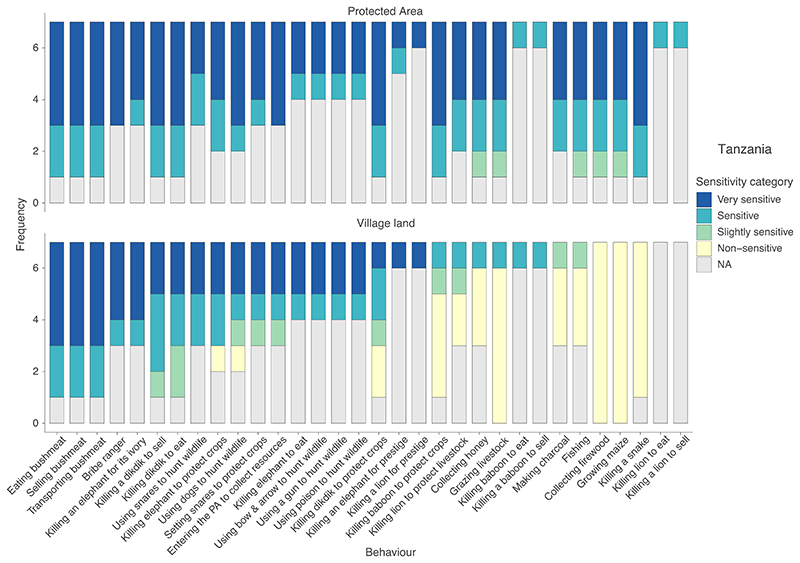
The reported sensitivity of behaviours when conducted in village/community land (bottom) and in protected areas (top) based on pile-sorts conducted by seven groups with 57 participants living in the Ruaha–Rungwa ecosystem in Tanzania. NA represents behaviours that participants reported they were unaware of, and thus were not able to classify.

In Tanzania, 57 participants in seven groups identified the 32 pile-sort cards into up to five categories of sensitivity (Figure 4). These were the same as those in Indonesia, but with the addition of a slightly sensitive category (where participants felt community members would discuss the topic but may not feel completely comfortable doing so).

Compared to Indonesia, a greater number of groups considered a greater number of behaviours as very sensitive or sensitive; in part this was due to illegality and potential repercussions, but also due to other factors (Supporting Information). When asked how topic sensitivity would change if behaviours were conducted inside protected areas, all groups that responded stated that topic sensitivity would increase (Figure 4), largely because protected area rules prohibit these activities.

### 3.4 Researcher reflections

In Indonesia, most people willing participated and shared their experiences of living alongside protected areas. However, in two groups we felt that participants were less willing to engage but were unsure if this was due to concerns about revealing information, or because the exercises were of less relevance as their villages were located further from protected areas. In Tanzania, throughout data collection, participants expressed interest, but also concern and sometimes suspicion. For example, one group stated that a mzungu (a Swahili phrase used to describe white people) had previously come to the community to conduct research on the National Park boundary, and that afterwards the boundaries were expanded. Two groups questioned the benefit of the research, highlighting that they had attended many research events but had never seen any change, nor experienced benefits. Other participants were cautious about why we were not collecting personal information such as their names and found this unusual despite our explanations that this was a protective measure. In some groups, we observed clear concerns from participants. For example, during one group exercise, participants repeatedly questioned our intentions, and were very hesitant to provide responses during the first free-list exercise. In another group, participants reported that no rule-breaking behaviour occurred and refused to sort cards into piles. In other groups, participants displayed apprehension, for example, by providing short answers, or warning others in the group not to reveal information. This discomfort highlights the importance of thinking carefully about how questions might be received, as well as the long-term legacy left by research.

## 4 Discussion

Understanding if, and why, a topic is sensitive is critical to the success of social science research (Lee & Renzetti, 1990; Sieber & Stanley, 1988), yet has received little attention in conservation. Our findings reveal substantial variation in the perceived sensitivity of different topics both within, and between different study contexts, highlighting the value of a mixed methods approach for understanding topic sensitivity.

### 4.1 Drivers of topic sensitivity

Overall, topics seemed considerably more sensitive in Tanzania, than Indonesia. All four behaviours investigated using our Sensitivity Index (the psychometric scale developed to assess topic sensitivity)in Tanzania were considered more sensitive than the most sensitive behaviour in Indonesia. Similarly, the pile-sort revealed that groups in Tanzania categorised a higher proportion of topics as very sensitive or sensitive, compared to groups in Indonesia. This difference in perceived sensitivity likely stems from a variety of factors, including differences in legislation, communities' awareness of the laws and differing levels of law enforcement, as well as varying cultural perspectives and norms regarding these behaviours (Atuo et al., 2020).

Generally, knowledge of conservation rules was higher among participants in Tanzania than Indonesia. For example, during group exercises in Tanzania participants often referenced Tanzanian law which deems that all wildlife belongs to the state; they described strict rules which prohibits the entering of National Parks or Game Reserves for any reason, and reported that if caught doing so, the likelihood of incurring sanctions was high. In Indonesia when known, awareness of rules was also a significant predictor of topic sensitivity: topics that were known to be prohibited were considered sensitive. In both country contexts, results suggest that when rules are well known and at least occasionally enforced, discussing noncompliant behaviour is likely to be sensitive. Contrastingly, in Indonesia, some behaviours which were illegal (e.g. hunting sambar) were openly discussed. This may be because poor knowledge of rules or low levels of enforcement meant participants associated less risk with discussing the behaviour. For example, in the Gola Forest, Liberia, research has found that when illegal behaviour is openly conducted and rules are not enforced, people are more willing to discuss rule-breaking (Jones et al., 2020).

Communities in Tanzania reported experiencing more challenges from living alongside protected areas than those in Indonesia, suggesting that any research was likely to be sensitive in these contexts because of the costs imposed on communities. Within Tanzania, the types of challenges reported differed across protected area types. For example, communities situated around Game Reserves often reported challenges relating to wildlife coexistence, including crop damage, livestock depredation and human fatalities. Discussions often detailed the nonmaterial burdens, such as grief, trauma and anxiety (Thondhlana et al., 2020) that communities experienced; in such instances conversations were sensitive because of the strong emotional responses they evoked (Lee & Renzetti, 1990). Communities living around Ruaha National Park often reported challenges associated with the way the National Park was managed, including how the law was enforced by rangers. In recent years, the eviction of villages and cattle herders from the former Usangu Game Reserve, as part of its incorporation into Ruaha National Park, has exacerbated communities’ resentment towards, and distrust of, government and protected areas (Walsh, 2012; Zia et al., 2011). Researchers working around other Tanzanian protected areas with similar environmental histories have found communities can perceive any research related to wildlife as a plot to further appropriate resources (Brockington et al., 2008; Weldemichel, 2020). Against a turbulent history, any research relating to protected areas is likely to be met with distrust and suspicion, and thus could be perceived as sensitive.

In Indonesia, communities’ also reported challenges associated with living alongside wildlife and protected areas, however, these were not reported as often by participants, and conversations did not evoke such strong emotional responses. While this may reflect cultural differences in how emotion is portrayed, it may also be an artefact of our sampling strategy and unequal coverage across the landscape. Recent research details the colonial militarisation of the Leuser Ecosystem and describes how communities in some areas were dispossessed of ancestral lands and conservation rules were imposed (Minarchek, 2019), thus discussions relating to conservation in other areas of the landscape may well be sensitive. In any research it is critical to understand and engage with the local environmental history, so that research can be designed and implemented appropriately.

### 4.2 Methods for measuring sensitivity

**Figure 5:**
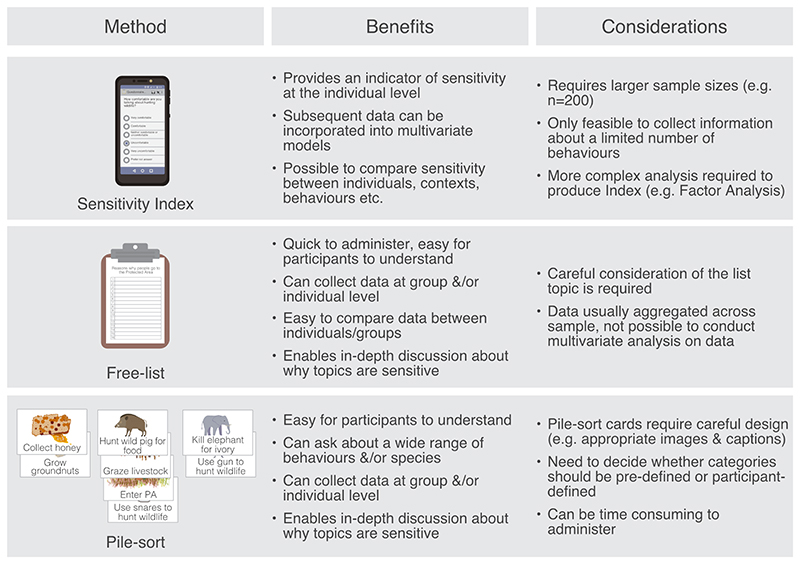
Summary of the benefits and design considerations of three methods (Sensitivity Index, free-lists and pile-sorts) tested to measure topic sensitivity.

We successfully present three new approaches to measure topic sensitivity (Figure 5). Applying our newly developed psychometric scale across two culturally different landscapes enabled us to test and verify performance in these locations. Symmetry of factor loadings across contexts suggests the resulting Sensitivity Index is reasonably robust. Overall, performance was stronger in Indonesia, than Tanzania, suggesting further refinement is needed, particularly if applied in other contexts. The variation in performance between contexts may be due to the lower variability in responses reported for items in Tanzania, or because of construct-underrepresentation, which can arise if items relevant to a latent variable are omitted (Furr, 2011). Refinement of item wording, and testing of additional questions, for example, items that measure descriptive norms (Cialdini, 2007) or respondent's acceptance of rules regarding different behaviours, could enhance the tool further. Creating a psychometric scale enabled measurement of topic sensitivity at the level of an individual respondent, however, scales usually require a significant amount of data to obtain sufficient statistical power. Indeed, there was a high degree of variation in the distribution of the sensitivity scores obtained, suggesting estimation of topic sensitivity using our Sensitivity Index may have benefited from larger samples. If resources are limited, employing this approach may not be feasible to inform wider study design. Promisingly, we found that by extracting responses from smaller subsets of individuals (e.g. 40 respondents) and crudely calculating the mean item response score, produced results reflective of the sophisticated Sensitivity Index (Supporting Information). This is reassuring, as it suggests our tool has potential to be adapted and tested in other contexts and easily deployed by conservationists to rapidly appraise topic sensitivity.

One limitation of psychometric scales and questionnaire-based research more broadly, is that their highly structured nature often leaves little flexibility to explore additional points of interest that arise. In contrast, both the pile-sort and free-list exercises provided freedom to ask about a wide range of behaviours, alongside valuable insights into why topics were sensitive, and how conservation rules were experienced, and perceived in the landscapes. Moreover, these methods are easy to use, require less resource and fewer participants, making them particularly attractive tools for familiarising oneself with the research context at the onset of research (Figure 5). A key limitation of any group exercise is that they run the risk of incurring biases, such as group think (members think similarly in order to maintain agreement) and halo effects (the status of one group member influences others; Nyumba et al., 2018). There is also debate about whether group exercises are appropriate settings to discuss sensitive topics, with careful consideration of the ethical implications of doing so required (Farquhar & Das, 1999). When conducting qualitive research that is less structured, it is also important to be aware that conversations can unintentionally transition into areas that can cause discomfort, requiring skilled facilitators that are properly prepared to handle sensitivity as and when it arises.

### 4.3 Who asks questions matters

In any research, who is conducting the research matters. Sensitivity may be affected by preconceptions held by participants about researchers and the power they hold, which in turn may influence their willingness to engage in research, and the information they choose to share (Blair et al., 2020). For example, in Tanzania, the presence of the lead researcher (a white European) was problematic for some communities, who associated research previously conducted by someone of a similar ethnicity, with evictions. Equally, a researcher's personal sense of identity influences the assumptions made about whether and why a topic is sensitive. As individuals we simultaneously belong to and identify with a range of groups (Farquhar & Das, 1999). Our conceptualisations of sensitivity are therefore informed by our experiences as a member of these groups, as well as the context in which the research is situated, with different norms more salient in different contexts (Farquhar & Das, 1999). Recognising sensitivity thus requires researchers to take a step back and to critically assess their own assumptions, to inwardly reflect on their own identity, to externally assess how these factors affect the research process and outcomes (Montana et al., 2020). Known as reflexivity, this process is increasingly promoted in conservation research (Beck et al., 2021; Montana et al.,2020; Satizábal et al., 2021), alongside practices that require researchers to consider their positionality, and the power-relations between themselves and participants (Attia & Edge, 2017; Satizábal et al., 2021). This is particularly important in a value-driven discipline such as conservation, where personal values risk influencing scientific objectivity (Brittain et al., 2020).

## 5 Conclusions

Few methods exist to measure topic sensitivity, meaning researchers and practitioners often rely on assumptions to design research. Our study highlights significant variation in the perceived sensitivity of topics both within, and across study contexts. What is sensitive in one context, may not be in another (Albaum et al., 2012), meaning it can be difficult in advance to assess how research will be perceived, and to determine the most appropriate methods to use to collect data and protect participants. Conservation research is increasingly conducted over large landscapes, where significant variation in perceptions will likely be encountered. Investing time and effort to obtain a robust understanding of topic sensitivity can inform better research. To this end, we encourage others to adapt and test our Sensitivity Index, within a mixed methods framework where resources allow, to make decisions on the suitability of methods (Nuno & St John, 2015) for researching topics that are potentially sensitive.

## Supplementary Material

Supplementary materials

## Figures and Tables

**Table 1:** Items used in the psychometric scale to measure individual’s perceptions of topic sensitivity. Respondents were asked about three behaviours in Indonesia (logging timber inside the National Park, clearing land in the National Park and hunting for wildlife on village land) and four in Tanzania (grazing livestock inside the nearest protected area, eating bushmeat, hunting wildlife on village land and entering the nearest protected area to collect resources). After piloting in Indonesia, the wording of two items (moral attitude and social desirability) was amended in line with respondents’ feedback.

Item	Wording	Likert response scales and scores
*Factor measuring behavioural approval*		
Injunctive norms surrounding the behaviour	If you did (behaviour), would your friends or family....	Strongly approve (1), approve (2), neutral (3), disapprove (4), strongly disapprove (5)
Moral attitude towards the behaviour	It is good to do (behaviour) (Indonesia only)	Strongly agree (1), agree (2), neutral (3), disagree (4), strongly disagree (5)
	It is wrong to do (behaviour) (Tanzania only)	Strongly agree (5), agree (4), neutral (3), disagree (2), strongly disagree (1)
Social desirability of the behaviour	If you did (behaviour), people in the community would think well of you *(Indonesia only)*	Strongly agree (1), agree (2), neutral (3), disagree (4), strongly disagree (5)
	If you did (behaviour), people in the community would think less of you *(Tanzania only)*	Strongly agree (5), agree (4), neutral (3), disagree (2), strongly disagree (1)
*Factor measuring willingness to talk*		
Personal comfort discussing the behaviour	If you did (behaviour), how comfortable would you feel answering questions honestly?	Very comfortable (1), comfortable (2), neutral (3), uncomfortable (4), very uncomfortable (5)
Community members’ willingness to discuss	How willing do you think people in the community would be to talk honestly about (behaviour)?	Very willing (1), willing (2), neutral (3), unwilling (4), very unwilling (5)

**Table 2:** Factor loadings and measures of model fit for two-factor exploratory factor analysis of our psychometric scale conducted for each country. Only factor loadings >0.4 are presented.

	Indonesia		Tanzania	
*Confirmation of factorability*				
Bartletts Test of Sphericity	1264.40, *p*-value = <0.001		979.31, *p*-value = <0.001	
KMO	0.81		0.69	
*Exploratory factor analysis loadings*	**Factor1**	**Factor2**	**Factor1**	**Factor2**
Injunctive norm (approval of friends/family)	0.56	–	0.40	–
Moral attitude towards behaviour	0.87	–	0.73	–
Social desirability of behaviour	0.61	–	0.85	–
Personal comfort discussing behaviour	–	0.57	–	0.66
Community willingness to discuss behaviour	–	0.78	–	0.54
Sum of squared loadings	1.59	1.07	1.44	0.84
Proportion variance	0.32	0.21	0.29	0.17
Cumulative variance	0.32	0.53	0.29	0.46
Proportion ratio	0.60	0.40	0.63	0.37
Number of observations	849		1025	
Likelihood chi-square	0.84 with prob<0.36		5.69 with prob<0.017	
RMSEA index	0.00 (90% CI: 0,0.08)		0.07 (90% CI: 0.02,0.13)	
Tucker-Lewis index	1.001		0.952	
SRMR	0.00		0.01	
BIC	-5.91		-1.25	
Factor1-Factor2 correlation	0.71		0.44	
*Tests for internal consistency*				
Cronbach’s alpha	0.76	0.69	0.73	0.52
Omega total	0.76	0.69	0.74	0.52

*Note: Cut-offs for good model fit:* Chi-square test of exact fit, root mean square error of approximation (RMSEA ≤ 0.06), Tucker-Lewis index (TLI?0.95), standardised root mean square residual (SRMR ≤ 0.08; Boateng et al., 2018), Bayesian information criteria (BIC < as possible).

**Table 3:** Log-odds regression coefficients with 95% confidence intervals from a beta mixed regression model, with random effects for respondent. The response represents a Sensitivity Index between 0 and 1. Text in bold represent p-values which had statistical significance of <0.05.

		Indonesia			Tanzania		
Predictors		Est	CI	*p*	Est	CI	p
(Intercept)		-0.41	-0.53 to -0.29	**<0.001**	1.17	0.74 to 1.61	**<0.001**
Gender^a^	Male	-0.14	-0.25 to -0.02	**0.020**	-0.08	-0.17 to 0.02	0.108
Age		0.03	-0.02 to 0.08	0.246	0.01	-0.03 to 0.06	0.557
Years of education		0.02	-0.03 to 0.07	0.377	0.01	-0.03 to 0.06	0.568
Behaviour^b^	Clearing land in NP	0.41	0.09 to 0.74	**0.012**		–	
	Logging in NP	0.52	0.05 to 0.98	**0.030**		–	
	Grazing livestock		–		-0.25	-0.93 to 0.42	0.465
	Eating bushmeat		–		-0.45	-0.95 t 0.06	0.083
	Entering the PA		–		-0.61	-1.20 to -0.02	**0.044**
Knowledge of rules^c^		0.31	0.16 to 0.45	**<0.001**	-0.07	-0.50 to 0.36	0.751
PA type^d^	Protected forest	0.32	-0.20 to 0.84	0.231		–	
	Game reserve		–		-0.04	-0.17 to 0.09	0.566
*Interactions*							
Knowledge of rules×clearing land		-0.07	-0.42 to 0.28	0.715		–	
Knowledge of rules×logging		0.39	-0.10 to 0.87	0.118		–	
Knowledge of rules×grazing livestock			–		0.01	-0.66 to 0.69	0.970
Knowledge of rules×eating bushmeat			–		0.26	-0.25 to 0.76	0.320
Knowledge of rules×entering the PA			–		0.31	-0.28 to 0.90	0.305
Protected Forest×land clearance		-0.15	-0.81 to 0.51	0.653		–	
Protected Forest× logging		0.19	-0.49 to 0.88	0.576		–	
Game Reserve×grazing livestock			–		0.13	-0.02 to 0.29	0.097
Game Reserve×eating bushmeat			–		-0.10	-0.25 -to 0.06	0.229
Game Reserve×entering PA			–		0.24	0.09 to 0.39	**0.002**
*Random effects*							
*σ* ^2^		-0.01			-0.02		
*τ*_00_		^0.07^id			0.08id		
ICC		1.14			1.36		
N		300id			281id		
Observations		829			979		
Marginal *R*^2^/conditional *R*^2^		0.782/1.031			0.192/1.288		

*Note:* Reference levels: ^a^Gender: female; ^b^Behaviour: Hunting on village land; ^c^No knowledge of rules regarding behaviour; ^d^Protected area type: National Park.

**Table 4:** Challenges of living alongside protected areas listed by participants during group exercises in Indonesia (10 groups, 60 participants) and Tanzania (eight groups, 66 participants), ordered by Smith’s Salience, with number groups mentioning an item (*n*). Items mentioned did not differ by gender, table combines results from both genders. NP indicates if the challenge was mentioned by groups living next to a National Park, Protection Forest (PF, Indonesia only) or Game Reserve (GR, Tanzania only).

Indonesia (10 items listed)	Salience	*n*	NP	PF	Tanzania (25 items listed)	Salience	*n*	NP	GR
Cannot expand farming areas	0.30	3	✓	✓	Crops destroyed by wildlife	0.69	7	✓	✓
Disturbance from wildlife	0.17	3	✓	✓	Livestock predated by wildlife	0.29	4		✓
Prohibited to grow crops in									
Protected Forest	0.10	1		✓	People injured/killed by wildlife	0.26	4		✓
Bear came to the village	0.10	1		✓	Conflicts over National Park boundaries	0.21	2	✓	
Monkeys raiding farms and houses	0.10	1		✓	People use arrest by rangers to threaten people	0.19	2	✓	
Unemployment	0.10	1		✓	Cannot access water sources	0.16	2	✓	
Cannot collect hardwood for house	0.08	1		✓	High fines if caught grazing livestock in the National Park	0.15	2	✓	
Crops destroyed by wildlife	0.05	1		✓	Movement of National Park boundary closer to the village	0.13	1	✓	
Border of PA is unclear	0.03	1	✓		Land shortages for agriculture	0.13	1	✓	
Landslides and floods from rivers	0.03	1		✓	Authorities will not allow electricity pylons through National Park to village	0.11	1	✓	
					Children not safe when wildlife is around	0.10	1		✓
					Nowhere to graze livestock	0.09	1	✓	
					Cannot collect firewood	0.08	1	✓	
					Corruption, having to pay rangers	0.06	1	✓	
					Tsetse flies	0.06	1		✓
					Land shortages increase conflicts between agriculturalists & pastoralists over grazing/cultivation land	0.05	1	✓	
					Rangers search houses, if they do not find anything they arrest or beat people	0.05	1	✓	
					Destruction of water sources by wildlife	0.05	1		✓
					People killed by rangers	0.05	1	✓	
					Poor relationship between National Park and community	0.03	1	✓	
					People/livestock lost after being chased by rangers	0.03	1	✓	
					Rangers do not inform village chief before making arrests	0.03	1	✓	
					Chased by buffalo	0.03	1		✓
					Unreliable infrastructure (due to remote location of village)	0.03	1		✓
					Livestock killed by rangers if found in the National Park	0.02	1	✓	

## Data Availability

Quantitative data are available open access: https://doi.org/10.6084/m9.figshare.22786229.
