# Supplementary material for: A mixed methods approach for measuring topic sensitivity in conservation
Source: People Nat (Hoboken). Author manuscript; Available in PMC 2023 Aug 9. (PMC7614907; doi:10.1002/pan3.10501)
Supplement: Supplementary materials [file EMS177416-supplement-Supplementary_materials.docx]

**Supplementary materials**

Table of Contents

[Appendix 1. Data collection 2](#_Toc129349532)

[Group exercise protocols – English 2](#_Toc129349533)

[Group exercise protocols – Bahasa Indonesia 6](#_Toc129349534)

[Group exercise protocols - Kiswahili 11](#_Toc129349535)

[Pile-sort behaviours 15](#_Toc129349536)

[Examples of A4 pile-sort cards (left Indonesia, right Tanzania) 17](#_Toc129349537)

[Example of datasheet used to collect data from group excercises and to make qualitative notes 18](#_Toc129349538)

[Survey Instrument – English and Bahasa Indonesia 23](#_Toc129349539)

[Survey Instrument – English and Kiswahili 33](#_Toc129349540)

[Appendix 2. Data analysis 43](#_Toc129349541)

[Descriptive statistics for response items measured with psychometric scale 43](#_Toc129349542)

[Exploratory Factor Analysis of psychometric scale data 45](#_Toc129349543)

[Indonesia 45](#_Toc129349544)

[Tanzania 47](#_Toc129349545)

[Sensitivity Index 49](#_Toc129349546)

[Creation of a composite Sensitivity Index 49](#_Toc129349547)

[Beta regression models 49](#_Toc129349548)

[Can simplistic analyses on smaller samples provide similar results to the Sensitivity Index? 50](#_Toc129349549)

[Free-listing analysis and results 51](#_Toc129349550)

[Calculating item salience 51](#_Toc129349551)

[Results from the first free-listing exercise (Reasons why people go to Protected Areas) 51](#_Toc129349552)

[Qualitative notes from second free-listing exercise (Challenges of living alongside PAs) 52](#_Toc129349553)

[Indonesia 52](#_Toc129349554)

[Tanzania 52](#_Toc129349555)

# Appendix 1. Data collection

## Group exercise protocols – English

Instructions to enumerators are highlighted in grey.

**Information script [15 minutes]**

Hello my name is *[name of enumerator]* and this is my colleague *[team member name].* We are researchers from *[name of partner institution]* working with Harriet Ibbett, who is from Bangor University in the UK. Her research aims to better understand the use of natural resources by people who live in the *[landscape name]*. We have asked you to join us today to ask for your help in better understanding your culture, and to identify how you think we should ask people about natural resource use. We expect this discussion to last around two and a half hours, however, it may be longer or shorter depending on your answers.

We are independent researchers, and are not related to the government or any NGOs, but we do have permission of the Indonesian government and the village authorities to carry out this research. Your participation is voluntary. You do not have to participant and you may stop participating at any time, without having to explain why. If you feel uncomfortable answering some of the questions, you do not have to answer. If you would like to skip a question or a topic, please say.

I *[name of enumerator]* will lead the discussion. I will ask questions and ask you to participate in exercises that we have prepared. *[Team member name]* here will record your responses. With your consent, we would like to record this discussion. This is because sometimes many people speak at once, and it can be hard to record every person’s opinion.

Your responses and discussion will be kept confidential and will not be linked to you individually or your village. The information you share with us will be stored safely, and we will not share the recordings with anyone else.

Harriet will use the results to help design future surveys on local communities’ natural resource use asks questions in ways that are polite and respectful in your culture. Harriet may also publish the findings internationally, to help researchers in other countries understand how best to research these topics.

We are here to learn about your community, we may have lots of questions, or there may be things we don’t understand, so we would be very grateful if you are happy to answer our questions. To make sure that everyone has an equal chance to speak and to share their thoughts and views, we would like to ask you to respect and listen to what each other has to say. There are no wrong or right answers, everyone will have a chance to speak.

This study has been approved by the ethics committee at Bangor University. If you have any concerns about this project, please speak to me and I will do my best to answer your query.

If you remain unhappy or wish to make a formal complaint, I can give you the contact details of someone to discuss this with.

There will be two activities.

The first will take around an hour, then we will have a short break.

The second will take around another hour.

**COVID-19 Precautions *[Used in Indonesia only – Tanzania fieldwork conducted pre-Covid)***

Before we start, we are aware that COVID is present in North Sumatra. We want to share with you the measures we are taking to reduce the risk of spreading COVID and to explain why we have asked you to sit like this.

Firstly, be assured that the research team is healthy and free from any obvious COVID-19 symptoms. If we start to display any team member starts to display symptoms we will isolate ourselves immediately.

To reduce risks, we would like for everyone to stay at least 2 metres apart at all times, this is why we have positioned the chairs like this.

Before we start, we ask you to wash your hands. We have also done so, this helps to reduce the spread of the disease.

We will also be wearing masks throughout the focus group, and ask you to wear one also. We will provide clean masks for you to keep. If you cannot hear, or need something repeating, please say.

All equipment has been cleaned, but to stop the spread, we ask that you do not touch it. Only I will touch the equipment we use. If you need to see the equipment more clearly, please say and I will help.

Thank you for your understanding in helping us to protect each other.

Do you have any questions or concerns so far?

(Pause here to give people time to think & comment)

Would you like to continue the meeting?

(Get verbal consent from every participant)

Would you be happy for us to record this meeting?

(Get verbal consent from every participant, if someone disagrees, do not record).

Go around the group and ask people for information about their age and ethnicity – explain this is for our research purposes – to help us understand the opinions of different groups that live in the village.

**Free-listing**  *[Estimated 1 hour]*

I would like you to think of all the reasons you know about why people who live in your community go to *[Protected area]*.

*[Pause to let people think]*

For example, people may go to collect resources, or for their livelihoods.

Many people may go for the same reason, or perhaps only a few people go for different reasons.

As a group, I would like you to tell me all the different reasons you know about why people go to the *[Protected area]*.

I would like you to tell me these one by one and we will list all the reasons down on a piece of paper.

[ Keep prompting ] Are there any other reasons you know why people go?

**List all items on large piece of paper in the order they are mentioned**

- Do they go for any natural resources?
- Are there culture or heritage reasons?
- Any other reasons?

**** BE CAREFUL NOT TO LEAD PEOPLE IN THEIR RESPONSES ****

Is that all?

Once the list is completed, for each activity ask:

- How many people do this activity? (e.g. everyone, very few)
- What types of people do this? (e.g. male, female, young / old )
- Why do people do this? (e.g. subsistence or income)
- When do people do this? How often do they go?

**Second free-list**

Now I want you to think of the ways in which you know that *[ Protected area]* negatively affects people who live in the community.

You may know of problems people face or challenges encountered.

Again, I would like you to tell me these, and one – by – one we will make a list and record it on paper.

**List all items on large piece of paper in the order they are mentioned**

- Are there things they would like to do but cant?
- Are there any costs from living near the protected area?
- Any more reasons?

When each activity is mentioned, ask the respondents to provide more information about this. E.g.

- How often does this happen?
- What do you do?
- Why is this a problem?

Are there any other reasons you know of?

**Third-free list**

Now I want you to think about the benefits of living alongside the Protected Area.

Please can you list them?

**List all items on large piece of paper in the order they are mentioned**

Ask the respondents to provide more information about each benefit.

Each if they say they get money from the park – how? Who gives them this? How is this managed? What do they use it for?

**Pile sorting [Estimate 1.5 hour ]**

For this next exercise, I have a pile of different cards. On each of these cards is a picture and a description of an activity someone might do in on land around the village.

As individuals, and community members, you may feel happier talking about some activities than others.

I would like you to look at each card, and as a group decide how happy you think people in this community would be to talk about this activity, if it was conducted on village land.

Different people might be more willing to talk about some activities than others, for different reasons.

For example, for some it may not be culturally appropriate to ask about a certain activity. For example, in the UK to ask a lady her age is very offensive.

Some things may be sensitive for other reasons.

If you say you do an activity you might worry others will disapprove or think worse of you.

Some activities may only be conducted in secret, or at night.

You might be worried discussing the activity will get you into trouble.

I would like you to look at each card, and sort them into different piles depending on how similar you think they will be for people around here to talk about.

For example, you may have one pile of cards showing activities that you think people will not like to talk about.

In another pile you may have cards showing activities that you think people will be very happy and have no problem to talk about.

**Go through each card on a one-by-one basis.**

- Get members to discuss the activity
- Ask them why is this activity sensitive / not sensitive?
- How the sensitivity of the activity contrasts with those in other piles (e.g. why is it more or less sensitive?)

**For species cards**

- Review all cards for the same species at the same time.
- Explore whether sensitivity changes if the reason for killing the animal changes.
- Record any other interesting information that arises about these activities (e.g. who does them, why, how often)

**** At the end of the pile sort - RECORD WHICH GROUP EACH CARD IS ALLOCATED TO****

Ask participants to name each pile – and provide a description about what this pile represents.

IF possible, take photos of the separate groups, and label each photo with the focus group card and the group number.

Next, looking carefully at these piles, would certain activities move pile if the activity was conducted in [Nearest PA]?

Do you think people would be more or less willing to talk about these activities?

Why?

**Wrap-up [10 minutes]**

Thank participants for coming.

Ask if they have any questions.

**At the end of the FG – photograph each list of paper, record the village and group number.**

**FAQs & Suggested Responses**

**What is the end goal of the research? What benefit will it bring to the community?**

- It is good for us as researchers to understand why people go to protected areas, and the problems they face regarding natural resource use.
- We can feed our findings back to the government about how communities need resources and their feelings towards protected areas.

**Why have we been separated by gender?**

- Men and women often use natural resources in different ways.
- By having groups of only men or only women, it helps us to get a deeper understanding of how this natural resource use differs.

**Last year, other researchers came and asked us similar questions. They promised this, yet nothing has happened or changed. Why should we talk to you now?**

- We are working here with the permission of the government & are required to feedback some of our findings.
- We can tell them your problems and the reasons why you feel you need to go to the PA.
- We can’t guarantee anything will change, but we can at least share your voices and concerns.

**In the village we also have problems with XXX.**

- Take notes about the problems, ask questions, show interest & concern.
- Say you are unable to make changes, but if you are able to share their concerns with the appropriate people you will.

**Access to research findings & dissemination results.**

- Results will be published in scientific reports.
- Results will also be used to inform a further study which will take place next year. We will return and complete that study.
- We are currently thinking about the best way to disseminate this information and would value your thoughts on how this can be achieved.

## Group exercise protocols – Bahasa Indonesia

**Information script [15 minutes]**

Salam (Assalammualaikum wr.wb atau Shalom atau horas). Nama saya xxx dan ini adalah teman saya xxx. Kami adalah peneliti dari Universitas Indonesia bekerjasama dengan Harriet Ibbett dari Universitas Bangor di Inggris. Penelitiannya bertujuan untuk mengetahui penggunaan sumber daya alam oleh masyarakat yang tinggal di sekitar kawasan Gunung Leuser. Kami mengundang BapakBapak atau Ibu-Ibu hari ini untuk membantu kami dalam memahami adat istiadat di sini, dan juga meminta pendapat dari BapakBapak atau Ibu-Ibu bagaimana kami bertanya mengenai cara masyarakat di sini memanfaatkan sumber daya alam. Harapannya, diskusi ini berlangsung tidak lama sekitar 2 – 2.5 jam, tapi kemungkinannya bisa lebih lama dan lebih cepat tergantung bagaimana Bapak atau Ibu menjawab.

Kami adalah peneliti independen, dan tidak bekerjasama dengan pemerintah ataupun NGO atau LSM, tetapi kami sudah memiliki izin dari pemerintah Indonesia dan dari apparat desa untuk melakukan penelitian ini. Keikutsertaan Anda adalah sukarela. Bapak atau Ibu boleh tidak mengikuti dan berhenti kapan saja tanpa menjelaskan alasannya. Jika Bapak atau ibu tidak nyaman untuk menjawab beberapa pertanyaan, maka tidak perlu dijawab. Jika Bapak atau Ibu ingin melewati pertanyaan atau topik, mohon beritahu kami.

Saya [nama] akan memimpin diskusi ini. Saya akan bertanya dan meminta Bapak/Ibu untuk berpartisipasi dalam kegiatan yang sudah kami persiapkan. XXX akan mencatat diskusi ini. Dengan persetujuan dari Bapak/Ibu, kami akan merekam diskusi ini, karena terkadang Bapak/Ibu berbicara bersamaan, sehingga akan sulit bagi kami untuk merekam setiap opini yang Bapak/Ibu sampaikan.

Jawaban dan hasil diskusi Bapak/Ibu akan dijaga kerahasiaannya dan tidak akan dilihat secara personal atau per desa. Informasi yang Bapak/Ibu berikan kepada kami akan tersimpan secara aman dan Kami tidak akan memberikan hasil diskusi kepada siapapun.

Harriet akan menggunakan hasil ini untuk merancang survei selanjutnya di masyarakat terkait penggunaan sumber daya alam yang ditanya dengan cara yang sopan dan sesuai dengan adat istiadat. Harriet juga akan memublikasikan hasil penelitian ini secara internasional agar dapat membantu peneliti-peneliti dari berbagai negara bagaimana cara terbaik melakukan penelitian dengan topik ini.

Kami di sini untuk belajar tentang komunitas/masyarakat di sini, kami mungkin akan menanyakan banyak pertanyaan atau mungkin ada hal yang tidak kami mengerti, karenanya kami sangat berharap Bapak/Ibu dengan senang hati menjawab pertanyaan-pertanyaan kami. Untuk memastikan setiap orang memiliki kesempatan yang sama dalam berbicara dan berpendapat, kami memohon setiap orang untuk mendengarkan dan menghargai apa yang orang lain sampaikan. Tidak ada jawaban salah atau benar, setiap orang memiliki kesempatan untuk berbicara.

Penelitian ini sudah disetujui oleh komite etik di Universitas Bangor, Inggris. Jika Bapak/Ibu ada hal-hal yang ingin ditanyakan terkait penelitian ini, silahkan untuk bertanya dan saya akan menjawab sebisa mungkin.

Jika ada yang masih kurang berkenan atau ada yang ingin ditanyakan, kami akan memberi kontak seseorang untuk diajak berdiskusi.

Akan ada dua kegiatan.

Kegiatan pertama akan memakan waktu sekitar 1 jam, kemudian kita akan istirahat sebentar.

Kegiatan kedua akan memakan waktu sekitar 2 jam.

Sejauh ini, apa ada yang ingin ditanyakan atau disampaikan?

(Pause here to give people time to think & comment)

Apakah Bapak/Ibu masih ingin melanjutkan diskusi ini?

(Get verbal consent from every participant)

Apakah Bapak/Ibu bersedia untuk kami merekam kegiatan ini?

(Get verbal consent from every participant, if someone disagrees, do not record).

Berkeliling grup untuk menanyakan peserta tentang umur dan suku – jelaskan bahwa ini adalah untuk kepentingan penelitian – membantu kami dalam memahami opini dari kelompok yang berbeda.

**Free-listing [Estimated 1 hour]**

Saya ingin Bapak/Ibu memberikan beberapa alasan mengapa masyarakat pergi ke [kawasan lindung].

Contohnya, masyarakat pergi untuk mencari sesuatu atau memasuki hutan untuk keperluan lain.

Banyak orang mungkin memiliki alasan yang sama, atau mungkin beberapa memiliki alasan yg berbeda.

Sebagai kelompok, Saya ingin Bapak/Ibu untuk memberitahu saya alasan-alasan yang berbeda mengapa masyarakat pergi ke [kawasan lindung].

Saya ingin Bapak/Ibu memberitahu saya satu persatu dan kami akan mencatat semua alasan secara berurutan di selembar kertas.

Apakah masih ada alasan lain?

**List all items on large piece of paper in the order they are mentioned**

- Apakah mereka pergi untuk mendapatkan sumberdaya alam?
- Apakah ada alasan budaya atau adat?
- Apakah ada alasan lain?

****JANGAN MENGARAHKAN JAWABAN MEREKA****

Apakah sudah semua?

Once the list is completed, for each activity, ask:

- Berapa banyak yang melakukan aktivitas ini? (contoh: semua orang, beberapa).
- Orang yang seperti apa yang melakukan aktivitas ini? (contoh: laki-laki, perempuan, muda/tua).
- Mengapa orang tersebut melakukan itu? (contoh: konsumsi sendiri atau pemasukan (dijual)).
- Kapan biasanya hal tersebut dilakukan? (Seberapa sering mereka pergi?)

**Second free-list**

Sekarang, saya ingin Bapak/Ibu menyampaikan dampak negatif dari taman nasional terhadap masyarakat yang tinggal di sekitar [kawasan lindung].

Bapak/Ibu mungkin juga mengetahui masalah atau tantangan yang dihadapi.

Sekali lagi, saya ingin Bapak/Ibu menyampaikan hal ini secara satu persatu, dan kami akan mencatat di kertas.

**List all items on large piece of paper in the order they are mentioned**

- Apakah ada kegiatan yang mereka ingin lakukan tetapi tidak bisa?
- Apakah ada dampak yang didapatkan karena tinggal di dekat kawasan lindung.
- Apakah ada alasan lain?

Ketika masing-masing kegiatan disebutkan, tanyakan kepada responden untuk mendapatkan informasi berikut

- Seberapa sering hal ini terjadi?
- Apa yang Anda lakukan?
- Mengapa hal ini menjadi masalah?

Apakah ada alasan lain?

**Third-free list**

Sekarang saya ingin Bapak/Ibu menyebutkan keuntungan yang didapat ketika tinggal di sekitar kawasan lindung.

Dapatkah Bapak/Ibu menyebutkannya?

**Catat semua pada kertas besar sesuai urutan yang mereka sebutkan.**

Tanyakan kepada responden untuk mendapatkan informasi lebih mengenai masing-masing keuntungan tersebut.

Jika mereka mengatakan mendapatkan uang dari Kawasan lindung – bagaimana? Siapa yang memberikan uang tersebut? Bagaimana hal tersebut diatur? Biasanya digunakan untuk apa uang tersebut?

**Pile sorting [Estimate 1.5 hour ]**

Pada kegiatan ini, saya memiliki beberapa kartu yang berbeda. Masing-masing kartu memiliki gambar dan penjelasan dari aktivitas yang mungkin dilakukan seseorang di sekitar desa.

Sebagai perseorangan dan anggota kelompok, Bapak/Ibu mungkin lebih senang menyampaikan tentang aktifitas dibandingkan yang lain.

Saya ingin Bapak/Ibu melihat setiap kartu, dan sebagai kelompok memutuskan apakah masyarakat akan senang menyampaikan tentang aktivitas tersebut jika kegiatan ini dilakukan di desa.

Beberapa orang mungkin lebih bersedia untuk menyampaikan tentang kegiatan tersebut dibandingkan yang lainnya untuk alasan lain.

Contohnya, mungkin secara kultur pertanyaan terkait beberapa kegiatan tidak pantas untuk ditanyakan. Seperti, di inggris, menanyakan umur pada seorang perempuan sangat tidak pantas.

Beberapa hal mungkin sensitif untuk alasan lain.

Jika Bapak/Ibu melakukan kegiatan yang mungkin dikhawatirkan akan membuat orang lain tidak setuju atau berpikir buruk tentang Bapak/Ibu.

Beberapa kegiatan mungkin hanya dilakukan secara rahasia atau di malam hari.

Bapak/Ibu mungkin akan khawatir jika membicarakan kegiatan tersebut akan membuat Bapak/ibu terkena masalah.

Saya ingin Bapak/Ibu melihat ke setiap kartu, dan mengelompokkan kartu tersebut berdasarkan bagaimana masyarakat akan membicarakannya.

Contohnya, Bapak/Ibu akan memiliki satu tumpuk kartu yang menunjukkan kegiatan yang mungkin masyarakat tidak akan suka untuk membicarakannya.

Di tumpukan lain, Bapak/Ibu mungkin akan memiliki kartu yang menunjukkan kegiatan yang mungkin masyarakat akan senang dan tidak memiliki masalah untuk membicarakannya.

**Go through each card on a one-by-one basis.**

- Diskusikan tentang kegiatan tersebut.
- Mengapa kegiatan tersebut sensitif/tidak sensitif.
- Bagaimana sensitivitas dari kegiatan tersebut berbeda dengan tumpukan yang lain (apakah lebih sensitif atau tidak)?

**For species cards**

- Tunjukan kartu dengan spesies yang sama secara bersamaan.
- Cari tahu apakah sensitivitasnya berubah ketika alasan untuk membunuh binatang tersebut berubah
- Catat informasi menarik yang muncul tentang kegiatan tersebut (contoh: siapa yang melakukannya, kenapa, seberapa sering).

** **Setelah responden mengelompokkan kartu** – **CATAT NOMOR GRUP DARI MASING-MASING KEGIATAN (KARTU)** **

Minta peserta untuk memberi nama pada setiap kelompok kartu – dan berikan deskripsi tentang kelompok tersebut.

Jika memungkinkan, ambil gambar untuk setiap kelompok kartu, dan beri label pada setiap foto dengan kode grup.

Selanjutnya, Saya ingin Bapak/Ibu untuk melihat kelompok kartu ini, apakah ada kegaitan yang ingin dipindahkan jika kegiatan tersebut dilakukan di [kawasan lindung]?

Menurut Bapak/Ibu apakah masyarakat bersedia untuk membicarakan tentang kegiatan-kegiatan ini?

Mengapa?

**Wrap-up [10 minutes]**

Terimakasih Bapak/Ibu atas partisipasinya.

Tanyakan apabila ada pertanyaan.

**Di akhir sesi Diskusi Kelompok – Ambil foto pada urutan di kertas, catat nama desa dan nomor grup.**

**FAQs & Suggested Responses**

**Apa tujuan akhir dari penelitian ini? Apa keuntungan yang masyarakat dapatkan?**

- Untuk mengetahui mengapa masyarakat pergi ke kawasan lindung dan masalah yang dihadapi terkait dengan pemanfaatan sumberdaya alam.
- Memberikan rekomendasi kepada pemerintah tentang bagaimana masyarakat membutuhkan sumberdaya dan pendapat mereka terhadap kawasan lindung.

**Mengapa dipisahkan berdasarkan jenis kelamin?**

- Laki-laki dan perempuan biasanya menggunakan sumberdaya alam secara berbeda.
- Dengan membuat kelompok yang hanya berisi laki-laki dan perempuan, akan membantu kami dalam memahami lebih lanjut bagaimana sumberdaya alam tersebut digunakan.

**Tahun lalu juga ada peneliti yang datang dan menanyakan hal yang sama. Mereka berjanji sesuatu, namun tidak ada perubahan. Untuk apa kami membicarakannya lagi kepada Anda?**

- Kami bekerja di sini dengan ijin dari pemerintah dan diwajibkan memberikan masukan dari hasil penelitian ini.
- Kami dapat memberitahukan mereka tentang masalah yang dihadapi dan alasan mengapa Bapak/Ibu merasa jika harus pergi ke kawasan lindung.
- Kami tidak bisa menjanjikan sesuatu akan berubah, tetapi setidaknya kami dapat menyampaikan suara/pendapat Bapak/Ibu.

**Di desa kami juga menemukan masalah dengan XXX**

- Mencatat tentang masalahnya, menanyakan pertanyaan, menunjukkan ketertarikan dan kekhawatiran.
- Katakan bahwa kamu tidak bisa melakukan perubahan, tetapi kamu dapat menyampaikan kekhawatirannya dengan orang yang pantas.

**Akses untuk mendapatkan hasil penelitian**

- Penelitian ini akan dipublikasi dalam laporan ilmiah.
- Hasil penelitian ini akan digunakan untuk dijadikan bahan pertimbangan pada penelitian selanjutnya yang akan dilakukan tahun depan.
- Saat ini kami sedang memikirkan cara terbaik untuk menyampaikan hasil penelitian ini.

## Group exercise protocols - Kiswahili

**Information script [15 minutes]**

Habari, Jina langu ni ……………. na huyu ni mwenzangu ……….. Ni watafiti tunaofanya kazi na Harriet Ibbett,ambaye ametokea Chuo Kikuu cha Bangor Uingereza. Utafiti wake una lengo la kuelewa namna rasilimali zinavyotumika na watu wanoishi katika uwanda wa Ruaha-Rungwa. Tumekuomba kuungana nasi leo ili utusaidie kuelewa utamaduni wenu na kutueleza namna sahihi ya kuuliza watu juu ya matumizi ya rasilimali asili. Tunatarajia mazungumzo kuchukua muda wa saa mbili, japokuwa yanaweza kuchukua muda mrefu au mfupi kutegemea na majibu yenu.

Sisi ni watafiti huru na hatuhusiani na Serikali au Mashirika yasiyokuwa ya serikali ila tuna kibali kutoka Serikali ya Tanzania na Serikali ya kijiji ya kufanya utafiti huu. Ushiriki wako ni wa kujitolea.Unaweza kujiondoa wakati wowote bila kutoa sababu. Kama hautajisikia vizuri kujibu baadhi ya maswali unarususiwa kutokujibu. Kama utataka kuruka swali au mada tafadhali sema.

Mimi {……..} nitaongoza majadiliano. Nitauliza maswali na kukuomba kushiriki katika mazoezi tuliyoyaandaa. XXX atamsaidia Harriet kuelewa kila mnachokisema , na Harriet ataandika majibu yenu na majadiliano anayoendelea kwenye daftari. Kwa ridhaa yako tutapenda pia kurekodi majadiliano haya. Hii ni kwa sababu wakati mwingine watu huongea kwa mara moja. Hivyo, kuwa vigumu kuandika maoni ya kila mtu.

Majibu yenu na majadiliano yatakuwa siri na hayatahusishwa na mtu mmoja mmoja au kijiji chenu. Taarifa mtakayotupatia itatunzwa kwenye kompyuta ambayo Harriet tuu ndio anauwezo wa kuitumia kwakutumia neno siri. Harriet atatumia majibu ya utafiti huu kusaidia kutengeneza tafiti za siku zijazo kwa jamii juu ya matumizi ya rasilimali kwa njia zinazozingatia upole na heshima kwa utamaduni wenu. Harriet pia atachapisha majibu ya utafiti kimataifa ili kusaidia watafiti wa nchi nyingine kuelewa jinsi ya kutafiti mada kama hizi.

Kwasababu tupo hapa kujifunza kuhusu jamii yenu tunaweza kuwa na maswali mengi au kunaweza kuwa na vitu hatuvielewi, hivyo tutashukuru sana kama utatujibu maswali yetu. Ili kuhakikisha kila mtu ananafasi sawa ya kuzungumza na kuchangia mawazo yao,tunakuomba uheshimu na usikilize kile ambacho kila mtu atasema. Hakuna majibu sahihi au yasiyosahihi na kila mmoja ananafasi sawa ya kuzungumza.

Utafiti huu umeruhusiwa na kamati ya maadili ya Chuo kikuu cha Bangor.Kama una swali lolote, tafadhali niulize na nitajitahidi niwezavyo kukujibu.

Kama utakuwa na wasiwasi au unataka kutoa malalamiko nitakupa mawasiliano ya mtu unayeweza kuzungumza naye.

Wasomee washiriki ratiba: zoezi 1 (kuorodhesha shughuli mbalimbali - kadirio la lisaa), mapumziko (dakika 10), halafu zoezi lingine (Kuweka shughuli kwenye makundi - kadirio la lisaa na nusu).

Je una swali lolote au wasiwasi mpaka sasa?

(Tafadhali wape watu nafasi ya kufikiri na kuchangia)

Uko tayari kuendelea na mazungumzo?

(Pata ridhaa ya maneno kutoka kwa kila mshiriki).

Utajisikia vizuri kama tutarekodi majadialiano haya

(Kama mtu yeyote hajakubali, tafadhali usirekodi).

Pita kwa kila mtu kwenye kundi na umwulize umri na kabila – elezea kuwa hii ni kwa ajili ya kutusaidia kuelewa maoni ya makundi mbali mbali ya watu yaliyomo kwenye kijiji.

**Kuorodhesha shughuli mbalimbali**  *[kadirio la lisaa]*

Ningependa ufikiri juu ya sababu zote unazojua zinazowafanya watu wanaoishi katika jamii yenu kwenda kwenye *[Hifadhi ya Taifa ya Ruaha / Pori la akiba(GR) / Pori tengefu(WMA)]*

*[ Subiri watu wafikiri ]*

Kwa mfano, watu huweza kwenda kuchukua rasilimali, au kwa ajili ya mahitaji yao ya kila siku.

Watu wengi wanaweza kwenda kwa sababu zile zile, au pengine ni watu wachache tu huwenda kwa sababu mbalimbali.

Kama kundi ningependa mniambie sababu tofauti tofauti inazowafanya watu waende kwenye *[ Hifadhi ya Taifa ya Ruaha / Pori la akiba (GR) / Pori Tengefu (WMA) ],* na kila mtakapotaja tutaandika kila moja kwenye karatasi.

(Endelea kuuliza) Unaweza kufikiri sababu zingine?

**Andika vitu vyote kwa kuzingatia mtiririko wa vinavyotajwa**

- Unaweza kupata rasilimali asili yoyote?
- Unaweza kupata rasilimali asili kwa ajili ya kipato?
- Je kuna sababu za kiutamaduni na urithi?
- Ni muhimu kwa imani yako?

****KUWA MAKINI USIWAPE WATU MAJIBU****

Ni hayo to?

Orodha hii ikikamilika, kama kuwinda, kuchunga mifugo, kuvua samaki, kurina asali au kuokota kuni yamejitokeza, uliza maswali:

- Watu wangapi wanafanya shughuli hizi?
- Watu gani hufanya shughuli hizi?
- Kwanini watu hufanya hivi/hufanya shughuli hizi?
- Ni wakati gani watu hufanya hivi/shughuli hizi?

**Second free list**

Sasa naomba ufikiri kwa namna unavyojua wewe kuhusu athari hasi za *[ Hifadhi ya Taifa ya Ruaha / Pori la akiba (GR) / Pori Tengefu (WMA)* kwa watu wanaoishi katika jamii

—kwa mfano unaweza kuwa unafahamu matatizo au changamoto ambazo watu hukutana nazo.

Tena, ningependa uniambie kuhusu changamoto hizo moja baada ya nyingine na tutatengeneza orodha na kuandika kwenye karatasi.

**Andika kwenye karatasi kubwa vitu vyote kwa kufuata mtiririko wa vinavyotajwa**

Vitu ambavyo unapenda kufanya, lakini hauruhusiwi, gharama za kuendesha maisha, afya na kipato

**Orodhesha vitu vyote kwenye karatasi kubwa kwa mtiririko wa vilivyo tajwa**

- Je kuna vitu ambavyo wangependa kufanya lakini hawawezi?
- Je kuna gharama zozote za kuishi pembezoni mwa hifadhi?
- Je kuna sababu nyingine zozote?

Je kuna sababu nyingine zozote unazozifahamu?

**Kuweka kwenye makundi [kadirio la lisa na nusu]**

Katika zoezi linalofuata, nina kadi tofauti tofauti. Katika kila kadi kuna picha za watu na ufafanuzi wa shughuli ambazo mtu anaweza kufanya katika ardhi ya kijiji na sababu za kufanya shughuli hizo.

Kama mtu binafsi na mwanajamii unaweza kujisikia huru kuzungumza kuhusu baadhi ya shughuli kuliko nyingine.

Ningependa muangalie kila kadi, na kama kundi, mfanye maamuzi kama watu watafurahi kiasi gani kuzungumzia shughuli tajwa kwenye kadi kama ikifanywa kwenye ardhi ya kjiji.

Vitu tofauti tofauti vinaweza kuwa nyeti kwasababu mbalimbali.

Baadhi ya vitu vinaweza kuwa nyeti kwasababu haikubaliki kiutamaduni au sio sahihi kuuliza kuhusu vitu hivyo. Kwa mfano, kwa Uingereza si sahihi kumwuliza mwanamke umri wake, Tanzania si sahihi, kumuuliza mtu kama ameenda chooni leo.

Vitu vingine vinaweza kuwa nyeti kwa sababu nyingine.

Kama ukifanya shughuli hizi unaweza kuwa na wasiwasi kuwa wengine hawata kuunga mkono au watakufikiria vibaya.

Au shughuli nyngine zinaweza kuwa zinafanyika kwa siri sana au usiku tuu.

Au unaweza kuwa na wasiwasi kujadili shughuli hiyo/hizo kunaweza kukuweka matatizoni.

Ningependa muangalie kila kadi, na mzichambue na kuziweka katika makundi kutegemea na vile zinavyoendana na jinsi watu wa jamii hii wanaweza kuzungumzia.

Kwa mfano, unaweza kuwa na fungu moja la kadi linaloonyesha shughuli ambazo mnafikiri watu hawatapenda kuzungumzia.

Katika fungu lingine unaweza kuwa na kadi zinazoonyesha shughuli ambazo unafikiri watu watafurahia sana na hawatakuwa na tatizo kuzungumzia.

**Pitia kila kadi kwa mfumo wa kuangalia mojamoja.**

- Waambie watu wafanye majadiliano juu ya shughuli
- Waulize kwanini shughuli hii ni nyeti/sio nyeti?
- Jinsi unyeti wa shughuli unavyotofautiana na zile zilizoko kwenye kundi jingine (m.f kwanini hii ni + au – nyeti?)

**Kwa kadi za wanyama**

- Rejea kila kadi kwa wanyama mara moja.
- Chunguza ikiwa unyeti unabadilika kama sababu ya kumuua mnyama inabadilika.

**** Mwishoni –ANDIKA JINA LA KILA KUNDI ZILIPOWEKWA KADI****

Waulize jina la kila kundi –na wakukumbushe sababu kwanini kadi ipo katika kundi hilo

[Kusanya kadi na zirudishe katika mpangilio sahihi. Rudia kuchambua kadi]

Halafu, ningependa kurudia kuchambua kadi kwenye makundi. Ila wakati huu ningependa mniambie jinsi mtakavyofurahia kuzungumzia shughuli zilizonyeshwa kwenye kadi kama zingefanywa ndani ya [RNP / GR / WMA].

Rejea kila kadi. Uliza kama watu bado wanafurahia kuzungumzia shughuli kwenye kadi ukilinganisha na kama ikifanyika kwenye eneo la kijiji.

**Wrap-up [10 minutes]**

Washukuru watu kwa kushiriki

Uliza kama kuna swali lingine

**Mwishoni mwa mjadala--piga picha kila orodha kwenye karatasi ya A2, andika jina la kijiji na namba ya kundi.**

**Maswali yanayojitokeza mara kwa mara na majibu yaliyopendekezwa**

**Je, lengo la mwisho la utafiti ni nini? Je, utaleta faida gani kwa jamii?**

- Ni vizuri sisi kama watafiti kuelewa ni kwa nini watu huenda kwenye maeneo yaliyohifadhiwa, na shida/ matatizo wanayokumbana nayo juu ya matumizi ya maliasili
- Tunaweza kutoa baadhi ya majibu ya utafiti huu kwa serikali kuhusu mahitaji ya jamii yanayowapelekea kuingia kwenye maeneo yaliyohifadhiwa ili kujipatia rasilimali

**Kwa nini tumetenganishwa kwa jinsia?**

- Kwa kawaida wanaume na wanawake hutumia rasilimali asili kwa njia tofauti
- Kwa kuwa na vikundi vya wanaume tu na wanawake tu, inatusaidia kupata ufahamu Zaidi wa jinsi utumiaji wa rasilimali asili hii hutofautiana .

**Mwaka jana, watafiti wengine walikuja na kutuuliza maswali kama hayo. Walitoa ahadi mbalimbali lakini hakuna kilichotokea/kubadilika. Kwanini tuzungumze na nyinyi sasa?**

- Tunafanya kazi hapa kwa idhini ya serikali na tunapaswa kutoa mrejesho wa baadhi ya majibu ya utafiti huu.
- Tunaweza kuwaambia matatizo yenu na sababu zinazowapelekea watu kufuata/kutafuta rasilimali ndani ya maeneo yaliyohifadhiwa.
- Hatuwezi kuahidi chochote kwa sasa lakini tunaweza kufikisha sauti zenu na mapendekezo yenu mahali husika.

**Katika kijiji hiki pia tuna shida na XXX**

- Andika maelezo juu ya shida zao, uliza maswali, onyesha shauku na wasiwasi.
- Hatuwezi kuahidi mabadiliko lakini tutawashirikisha wahusika matatizo yenu.

**Upatikanaji wa matokeo ya utafiti na namna yatakavyosambazwa.**

- Matokeo yatachapishwa katika ripoti ya kisayansi
- Matokeo yatatumika pia kuarifu uchunguzi Zaidi ambao utafanyika mwakani. Tutarudi kwa lengo la kumalizia utafiti huu.
- Hivi sasa tunafikiria juu ya njia bora ya kusambaza taarifa hii na tunathamini mawazo yenu juu ya jinsi tunavyoweza kufanikiwa katika hili.

## Pile-sort behaviours

Table S1a. Descriptions and translations of the behaviours depicted on cards used during pile sort activities.

| **Behaviours in Indonesia** | | | **Behaviours in Tanzania** | | |
| --- | --- | --- | --- | --- | --- |
| **English** | | **Bahasa Indonesia** | **English** | | **Kiswahili** |
| 1 | Growing rice | Menanam padi | 1 | Grow maize | Kulima mahindi |
| 2 | Fishing | Mencari ikan | 2 | Fishing | Uvuvi |
| 3 | Clearing land for palm oil | Membuka iahan untuk kebun sawit | 3 | Collecting wood | Kuokota kuni |
| 4 | Collecting firewood | Mengumpulkan kayu bakar | 4 | Killing dikdik for stew | Huua digi digi kwa ajili ya kitoweo |
| 5 | Growing corn | Menanam jagung | 5 | Killing dikdik for sale | Huua digi digi kwa ajili ya kuuza |
| 6 | Collecting plants for medicine | Mengumpulkan tanaman untuk obat | 6 | Killing dikdik to protect crops | Huua digi digi kwa ajili ya kuzuia wasiharibu mazao |
| 7 | Cutting trees for money | Menebang pohon yang bernilai jual tinggi | 7 | Feeding/herding livestock | Kulisha/kuchunga mifugo |
| 8 | Growing fruit trees | Menanam pohon buah-buahan | 8 | Honey | Kurina asali |
| 9 | Collecting rattan | Mencari rotan | 9 | Eating bushmeat | Kula nayampori |
| 10 | Grazing cows | Menggembala ternak | 10 | Selling bushmeat | Kuuza nyamapori |
| 11 | Collecting honey | Mengambil madu | 11 | Transporting bushmeat | Kusafirisha nyamapori |
| 12 | Collecting wild birds | Mengambil burung liar | 12 | Making charcoal | Kuchoma mkaa |
| 13 | Cut trees for house | Menebang pohon untuk rumah | 13 | Killing elephant for stew | Kuua tembo kwa ajili ya kitoweo |
| 14 | Kill wild pig to sell | Membunuh babi liar untuk dijual | 14 | Killing elephant to sell their ivory | Kuua tembo kwa ajili ya kuuza meno yao |
| 15 | Kill wild pig for food | Membunuh babi liar untuk dimakan | 15 | Killing elephant to prevent crop damage | Kuua tembo ili kuzuia wasibaribu mazao |
| 16 | Kill wild pig to protect farms | Membunuh babi liar untuk melindungi kebun | 16 | Killing elephants for prestige | Kuua tembo kwa ajili ya ufahari |
| 17 | Kill a snake | Membunuh ular | 17 | Killing snake | Kuua nyoka |
| 18 | Kill elephant to sell | Membunuh gajah untuk dijual | 18 | Killing monkeys for stew | Kuua nyani kwa ajili ya kitoweo |
| 19 | Kill elephant to protect farms | Membunuh gajah untuk melindungi kebun | 19 | Killing monkeys for sale | Kuua nyani kwa ajili ya kuuza |
| 20 | Kill monkeys to eat | Membunuh monyet untuk dimakan | 20 | Killing monkeys to prevent crop damage | Kuua nyani kwa ajili ya wasiharibu mazao |
| 21 | Kill monkeys to protect farms | Membunuh monyet untuk melindungi kebun | 21 | Setting snares to protect crops | Kutega nyaya kwa ajili ya kulinda mazao |
| 22 | Kill pangolin to eat | Membunuh trenggiling untuk dimakan | 22 | Setting snares to catch & kill wildlife | Kutega nyaya kwa ajili ya kukumata na kuua wanyamapori |
| 23 | Kill pangolin to sell | Membunuh trenggiling untuk dijual | 23 | Hunting with a gun | Kuwinda kwa kutumia bunduki |
| 24 | Kill orangutan to protect crops | Membunuh orangutan untuk melindungi kebun | 24 | Using poison to kill wildlife | Kutumia sumu kuua wanyampori |
| 25 | Kill orangutan for money | Membunuh orangutan untuk dijual | 25 | Using bow & arrow to hunt wildlife | Kutumia mshale na upinde kuwinda wanyampori |
| 26 | Kill sambar for food | Membunuh rusa untuk dimakan | 26 | Hunting using dogs | Kuwinda kwa kutumia mbwa |
| 27 | Kill sambar to sell | Membunuh rusa untuk dijual | 27 | Killing lion to sell | Kuua simba kwa ajili ya kuuza |
| 28 | Kill sambar to protect farms | Membunuh rusa untuk melindungi kebun | 28 | Kill lions to protect livestock | Kuua simba kwa ajili ya luinda mifugo |
| 29 | Kill tiger to sell | Membunuh harimau untuk dijual | 29 | Killing lion for stew | Kuua simba kwa ajili ya kitoweo |
| 30 | Kill tiger to protect the village | Membunuh harimau untuk melindungi desa | 30 | Killing lion for prestige | Kuua simba kwa ajili ya ufahari |
| 31 | Using snares around the farm | Menggunakan jerat di sekitar kebun | 31 | Bribe wildlife officer | Kumpa rushwa askari wa wanyamapori |
| 32 | Using snares in the forest | Menggunakan jerat di hutan | 32 | Enter the protected area to collect resources | Kuingia ndani ya hifadhi kuokota rasilmali |
| 33 | Using rifles to kill wildlife | Menggunakan senapan untuk membunuh satwa liar |  |  |  |
| 34 | Using slingshot to kill wildlife | Menggunakan katapel untuk membunuh satwa liar |  |  |  |
| 35 | Using dogs to catch wildlife | Menggunakan anjing untuk menagkap satwa liar |  |  |  |
| 36 | Use poison to kill wildlife | Menggunakan racun untuk membunuh satwa liar |  |  |  |
| 37 | Bribing forest rangers | Menyuap polisi hutan |  |  |  |

## Examples of A4 pile-sort cards (left Indonesia, right Tanzania)

|  |  |
| --- | --- |
|  |  |

## Example of datasheet used to collect data from group excercises and to make qualitative notes

| Date: |  | |
| --- | --- | --- |
| Focus group facilitator:*(circle)* |  | |
| Focus group recorder:*(circle)* |  | |
| Focus group no (ID):  *(e.g. V#1_M for males in 1^st^ village)* |  | |
| Focus group type: | All male / All female | |
| Region: |  | |
| District: |  | |
| Subdistrict: |  | |
| Village name |  | |
| Nearest type of Protected Area: | Gunung Leuser National Park / Protected forest / other: | |
| Start time: |  | FG Duration: |
| End time: |  | hrs |

| Participant number | Gender | Approximate age | Ethnicity | Consent… | | |
| --- | --- | --- | --- | --- | --- | --- |
|  |  |  |  | To participate | To record | To photo |
| 1 |  |  |  |  |  |  |
| 2 |  |  |  |  |  |  |
| 3 |  |  |  |  |  |  |
| 4 |  |  |  |  |  |  |
| 5 |  |  |  |  |  |  |
| 6 |  |  |  |  |  |  |
| 7 |  |  |  |  |  |  |
| 8 |  |  |  |  |  |  |
| 9 |  |  |  |  |  |  |
| 10 |  |  |  |  |  |  |

**Exercise 1 - Free-list**

*It is very important you record both the activity and the order in which it was mentioned.*

Keep listing items until the options are exhausted.

| **List 1 – Reasons why people go to TNGL** | | **List 2 – negative impacts of TNGL** | |
| --- | --- | --- | --- |
| 1 |  | 1 |  |
| 2 |  | 2 |  |
| 3 |  | 3 |  |
| 4 |  | 4 |  |
| 5 |  | 5 |  |
| 6 |  | 6 |  |
| 7 |  | 7 |  |
| 8 |  | 8 |  |
| 9 |  | 9 |  |
| 10 |  | 10 |  |
| 11 |  | 11 |  |
| 12 |  | 12 |  |
| 13 |  | 13 |  |
| 14 |  | 14 |  |
| 15 |  | 15 |  |
| **List 3 - Benefits of Protected Area** | | | |
| 1 |  | 7 |  |
| 2 |  | 8 |  |
| 3 |  | 9 |  |
| 4 |  | 10 |  |
| 5 |  | 11 |  |
| 6 |  | 12 |  |

**LIST 1 ONLY - REASONS WHY PEOPLE GO TO THE PROTECTED AREA**

Use this space to record information that arises during the focus group about different activities.

| **Activity 1:** |
| --- |
| *E.g. What proportion of people do this? (e.g. everyone, very few). Who does this? (age, gender, ethnicity) When? Where? How often?*  *Why?* |
| **Activity 2:** |
| *E.g. What proportion of people do this? (e.g. everyone, very few). Who does this? (age, gender, ethnicity) When? Where? How often?*  *Why?* |
| **Activity 3:** |
| *E.g. What proportion of people do this? (e.g. everyone, very few). Who does this? (age, gender, ethnicity) When? Where? How often?*  *Why?* |
| **Activity 4:** |
| *E.g. What proportion of people do this? (e.g. everyone, very few). Who does this? (age, gender, ethnicity) When? Where? How often?*  *Why?* |

***Repeated for all activities listed.***

| **ADDITIONAL INFORMATION COLLECTED FROM OTHER FREE- LISTS** |
| --- |
|  |

**Pile sorting datasheet**

Record the reason given for the pile each card was sorted into. Wait until the end of the exercise to record the pile – as cards may change piles throughout the exercise.

| **Card** | | **1^st^ sort (non PA)** | **Reason why card is in this pile**  **(Outside PA)** | **2^nd^ sort (PA)** | **Reason why card is in this pile**  **(Inside PA)** |
| --- | --- | --- | --- | --- | --- |
| **1** | Growing rice |  |  |  |  |
| **2** | Fishing |  |  |  |  |
| **3** | Clearing land for palm oil |  |  |  |  |
| **4** | Collecting firewood |  |  |  |  |
| **5** | Growing corn |  |  |  |  |
| **6** | Collecting plants for medicine |  |  |  |  |
| **7** | Cutting trees for money |  |  |  |  |

Repeated for all behaviours asked about

**Pile Names**

*Complete this sheet once all the cards have been sorted into piles, but before the piles are reassigned according to Protected Area sensitivity*

| **Pile descriptions – Activities conducted in village lands** | | |
| --- | --- | --- |
| **Pile No.** | **Pile name** | **Pile description**  *Brief description of the reason WHY these items are grouped together* |
|  |  |  |
|  |  |  |
|  |  |  |
|  |  |  |
|  |  |  |
|  |  |  |
|  |  |  |

## Survey Instrument – English and Bahasa Indonesia

Table S1b. Survey instrument used in Indonesia to collect data using ODK.

|  | **Question::English** | **Instruction/prompt::English** | **Question::Indonesian** | **Instruction/prompt::Indonesian** |
| --- | --- | --- | --- | --- |
| Section 1. Survey location | | | | |
| start_time |  |  |  |  |
| end_time |  |  |  |  |
| Date |  |  |  |  |
| enumerator | Who is conducting the interview? | Siapa yang melakukan wawancara? |  |  |
| region | Province | Area |  |  |
| district | District | Kabupaten |  |  |
| district_other | If other district, please write | Jika Kabupaten lain, silahkan ditulis |  |  |
| subdistrict | Sub-district | Kecamatan |  |  |
| subdistrict_other | if other subdistrict, please write | Jika Kecamatan lain, silahkan ditulis |  |  |
| village | Name of the village | Nama Desa |  |  |
| subvill | Name of the sub-village | Nama Dusun |  |  |
| pa_type | Nearest Protected Area | Kawasan lindung terdekat | **Do not ask the respondent this** | **Jangan tanyakan ini kepada responden** |
| pa_type_other | If other, name of the Protected Area | Jika ada yang lain, berikan nama kawasan lindungnya |  |  |
| Section 2. Participant consent | | | | |
| ethic | Research ethics | Etika penelitian |  |  |
| ethics_statement | Read the Consent Script to the participant | Membaca naskah persetujuan kepada peserta | Hello. My name is ${enumerator}, I am a researcher from the University of Indonesia, and I am helping Harriet Ibbett, who is from Bangor University in the UK to conduct research. Harriet’s research is all about understanding the best way to ask questions about natural resource use.  The survey has two parts. First of all, we will ask your opinion about different types of natural resource use. The second part of the survey is to find out how you most prefer to answer questions about an activity that might be considered sensitive.   We are asking lots of people to complete this survey so that we can understand how people prefer to answer questions. Using your opinions and suggestions we will design a new survey, which will involve collecting information from local people about resource use around Protected Areas. The information you provide us is very important in making sure that we ask questions in the right way.   The questionnaire will take about 1hour 15 minutes to complete. Any information you provide will be anonymous, this means I will not record your name, or any information that can personally identify you or your household. We may tell people your answers, but we will not reveal that you gave the information. I will record all your answers on this phone. All your answers will then be saved on a secure computer which can only be accessed by Harriet using a password.   At the end of the research, Harriet will write a report on her findings. This report will be used to help other researchers conduct research that better meets the needs of local people. Some results may also be published internationally so that other people in different countries can learn from our experience working with communities here.  Please note that we are independent, we are not related to the government or any NGOs and we have neutral views. We have permission of the Indonesian government and the village chief to carry out this research. However, participation is voluntary. You do not have to participant and you stop participating at any time, without explanation. If you do I will discard your responses. If you feel uncomfortable answering some of the questions, you do not have to answer. If you would like to skip a question or a topic, please say.  This study has been reviewed by, and received ethics clearance through Bangor University. If you have any questions, please ask me and I will do my best to answer them.   If you remain unhappy or wish to make a formal complaint, I can give you the contact details of someone to discuss this with. | Halo nama saya ${enumerator}, saya peneliti dari Universitas Indonesia dan saya akan membantu Harriet Ibbett dari Universitas Bangor di Inggris untuk melakukan penelitian. Penelitiannya tentang memahami cara terbaik dalam menanyakan pertanyaan tentang penggunaan sumberdaya alam.  Survei ini terdiri dari 2 bagian. Bagian pertama, kami akan menanyakan opini Bapak/Ibu tentang berbagai jenis penggunaan sumberdaya alam. Bagian kedua, untuk menemukan cara bagaimana Bapak/Ibu paling nyaman menjawab pertanyaan tentang kegiatan yang dianggap sensitif.  Kami menanyakan banyak orang untuk melengkapi survei ini, sehingga kami bisa memahami bagaimana orang menjawab pertanyaan. Berdasarkan opini dan saran dari Bapak/Ibu, kami akan merancang survei baru, yang akan melibatkan pengumpulan informasi dari masyarakat lokal terkait pemanfaatan sumberdaya alam di kawasan lindung. Informasi yang Bapak/Ibu berikan sangat penting bagi kami sehingga kami bisa menanyakan pertanyaan secara tepat.  Kuesioner ini akan memakan waktu sekitar 1 jam 15 menit. Informasi yang Bapak/Ibu berikan bersifat anonim, yang artinya kami tidak akan mencatat nama, atau informasi personal terkait rumah tangga Bapak/Ibu. Kami kemungkinan akan memberikan jawaban Bapak/Ibu ke pihak lain, tetapi tidak akan memberitahukan dari siapa kami mendapat informasi tersebut. Saya akan merekam jawaban Bapak/Ibu di hp ini. Semua jawaban akan disimpan pada komputer yang aman yang hanya bisa di akses oleh Harriet dengan password.  Di akhir penelitian, Harriet akan menulis laporan berdasarkan penemuan di lapangan. Laporan ini akan dipakai untuk membantu peneliti-peneliti lain dalam melakukan penelitian sehingga bisa lebih sesuai dengan kebutuhan masyarakat lokal. Beberapa hasil akan dipublikasikan secara internasional sehingga masyarakat dari berbagai negara bisa belajar dari pengalaman kita bekerja dengan komunitas di sini.  Perlu diketahui bahwa kami independen, kami tidak ada kaitannya dengan pemerintah atau lembaga lainnya dan kami memiliki pandangan yang netral. Kami sudah mendapatkan izin dari pemerintah Indonesia dan juga kepala desa untuk melakukan penelitian ini. Namun demikian, keikutsertaan Bapak/Ibu bersifat sukarela.   Bapak/ibu tidak perlu berpartisipasi atau berhenti kapan saja tanpa alasan. Jika Bapak/Ibu melakukan itu, saya akan menghapus jawaban Bapak/Ibu. Jika Bapak/Ibu merasa tidak nyaman untuk menjawab beberapa pertanyaan, maka Bapak/Ibu tidak perlu menjawabnya. Jika Bapak/Ibu ingin melewati pertanyaan atau topik, mohon sampaikan kepada saya.  Penelitian ini sudah ditinjau dan juga telah mendapatkan persetujuan etik dari Universitas Bangor. Jika Bapak/Ibu memiliki pertanyaan, mohon sampaikan kepada saya, dan saya akan berusaha untuk menjawab sebaik mungkin.  Jika Bapak/Ibu masih merasa kurang senang atau ingin menyampaikan keluhan, saya bisa memberikan kontak kepada seseorang untuk menyampaikan hal tersebut. |
| consent | Did participant give their consent to participate? | Apakah peserta memberikan izin untuk berpartisipasi? |  |  |
| no_consent | Thank the participant and end the survey. | Ucapkan terimakasih dan mengakhiri survei. |  |  |
| consent_gender | What was the gender of the participant? | Apa jenis kelamin peserta? |  |  |
| Section 3. Covid precautions | | | | |
| covid | Covid | Covid |  |  |
| covid_note | Reminder: COVID Precautions | Pengingat: Tindakan pencegahan COVID-19 | If the guide has not already explained our COVID precautions then explain to the respondent:  1. That the team are clear of symptoms  2. That we will be working outside and maintaining social distancing  3. We will be washing hands frequently 4. That we wear masks to protect ourselves and the respondent | Jika pemandu belum menjelaskan tentang cara kita mewaspadai COVID maka jelaskan kepada responden:   1. Bahwa tim sudah bebas dari semua gejala. 2. Bahwa tidak akan bekerja di luar dan menjaga jarak.  3. Akan membasuh tangan sesering mungkin. 4. Bahwa kita mengenakan masker untuk melindungi diri sendiri dan responden. |
| covid_symptoms | Does anyone in your household have symptoms of COVID19 that have developed over the previous week? | Apakah ada anggota rumah tangga Bapak/Ibu yang terkena COVID19? | These are:  • a new and persistent cough • difficulty in breathing • a high fever, • a recent loss of taste or smell | Adalah: • batuk yang baru dan terus menerus • kesulitan bernapas • demam tinggi • kehilangan indera perasa dan penciuman |
| covid_yes | Thank the respondent for their time, explain that even though the sick individual may not have COVID we do not want to put other respondents at risk if they do have COVID. Wish them or their household member a quick recovery | Berterimakasih pada responden untuk waktunya, jelaskan bahwa walaupun orang yang sakit mungkin bukan karena COVID, tapi kami tidak ingin responden yang lain berisiko jika mereka terkena COVID. Katakan semoga anggota rumah tangga yang sakit lekas sembuh. |  |  |
| covid_mask | Wearing disposable masks | Kenakan masker yang bisa didaur ulang. | Would you like me to provide a mask for you to wear also? | Untuk melindungi saya dan Bapak/Ibu, Jika Bapak/Ibu bersedia, saya bisa memberikan masker untuk dipakai oleh Bapak/Ibu. |
| Section 4. Participant demographics | | | | |
| demographics | Participant demographics | Demografi peserta |  |  |
| gender | What gender is the participant? | Apa jenis kelamin peserta? |  |  |
| age | How old are you? | Berapa umur Anda? | If unknown, ask them to estimate their age | Jika tidak tahu, tanyakan perkiraan umurnya. |
| ethn | Ethnicity | Suku apa Anda? |  |  |
| ethn_other | Please specify which ethnic group | Tolong dispesifikasi suku Anda |  |  |
| language | What is the main language you speak? | Apa bahasa yang sering digunakan? |  |  |
| language_other | Please specify which language | Tolong dispesifikasi bahasa Anda |  |  |
| religious | Are you religious? | Apakah Anda meyakini suatu agama? |  |  |
| religion1 | Participants Religion | Agama peserta |  |  |
| religion | What is your religion? | Apa agama Anda? |  |  |
| religion_other | Please specify which religion | Tolong dispesifikasi agama Anda |  |  |
| religion_importance | How important is religion to you? | Seberapa penting agama bagi Anda? |  |  |
|  |  |  |  |  |
| yrs_ed | How many years of schooling do you have? | Berapa tahun Anda sekolah? | Enter approximate number of years (max 12 years)  If none, enter 0  For university, write 12 plus number of years of university completed e.g. 12 + 4 years = 16years | Masukan perkiraan jumlah tahun.  Jika tidak sekolah, masukan 0  Untuk setiap tahun pendidikan tinggi, tambahkan 1,contoh 4 tahun kuliah = 12 + 4 = 16. |
| literacy | Can you read? | Apakah Anda bisa membaca? |  |  |
| literacy_ease | How do you find reading? | Seberapa mudah membaca untuk Anda? | Read out options to respondent | Bacakan opsi kepada responden. |
| birth_month | Do you know the month in which you were born? | Apakah Anda tahu di bulan apa Anda lahir? |  |  |
| month | Birth month | Bulan lahir |  |  |
| own_mobile | Do you personally own a mobile phone? | Apakah Anda memiliki telepon genggam sendiri? | This means the individual. Not the household. | Untuk individu, bukan rumah tangga atau keluarga. |
| know_pa | Do you know the name of the nearest Protected Area? | Apakah Anda tau nama dari kawasan lindung terdekat di sekitar sini? |  |  |
| pa_name | Name of the nearest Protected Area | Nama dari kawasan lindung terdekat |  |  |
| pa_other | If other, name of the Protected Area | Jika ada yang lain, berikan nama kawasan lindungnya |  |  |
| Section 5. Sensitive behaviour 1 – Clearing land | | | | |
| land | Clearing forest | Membuka hutan |  |  |
| sens_note | Measuring sensitive behaviours | Mengukur kegiatan sensitif | I will now ask you about a range of activities that you, members of your household, other family members, your friends or neighbours in the village might do.   For each activity, I will ask several questions. I don’t want to know whether you do these activities, but just about your opinions.  The first activity is clearing forest. | "Sekarang saya akan bertanya tentang beberapa kegiatan yang Anda, anggota keluarga Anda, anggota keluarga lainnya, teman Anda atau tetangga di desa mungkin lakukan.  Untuk setiap kegiatan, saya akan menanyakan beberapa pertanyaan. Saya tidak ingin mengetahui apakah Anda melakukan kegiatan tersebut, tetapi hanya pendapat Anda tentang kegiatan tersebut.  Kegiatan pertama adalah membuka hutan" |
| comm_land | If we ask people in the community if they **clear forest** inside TNGL, how willing do you think people will be to talk to us honestly about this? | Jika kami bertanya masyarakat di kelompok jika mereka **membuka hutan** di dalam TNGL, menurut Anda seberapa bersedia mereka untuk berkata jujur tentang itu ? |  |  |
| approval_land | If you personally cleared forest in the TNGL, do you think your friends & family would... | Jika Anda membuka hutan di TNGL, menurut Anda keluarga dan teman Anda akan…. |  |  |
| morals_land | To what extent do you agree with the following statement? | Sejauh mana Anda setuju dengan pernyataan ini? | *"It is acceptable to clear forest in the TNGL"* | *"Memembuka hutan di TNGL dapat diterima"* |
| sdb_land | To what extent do you agree with the following statement? | Sejauh mana Anda setuju dengan pernyataan ini? | *"If I cleared forest in the TNGL, people in the community would think well of me"* | *"Jika saya membuka hutan di TNGL, anggota masyarakat akan berpikir baik tentang saya"* |
| comfort_land | If you clearing forest inside the TNGL and a researcher asked you questions about that, how comfortable would you feel to answer questions truthfully? | Jika Anda membuka hutan di TNGL, dan peneliti menanyakan pertanyaan tentang hal tersebut, seberapa nyaman anda akan menjawab pertanyaan tersebut secara jujur? |  |  |
| comwhy_land | Why would you be uncomfortable dicussing clearing forest in the TNGL? | Mengapa anda merasa tidak nyaman untuk membicarakan tentang membuka hutan di TNGL? |  |  |
| rules_land | Are there any rules about clearing forest in the TNGL? | Apakah ada aturan tentang membuka hutan di TNGL? |  |  |
| senscale_land | Explain the Sensitivity Scale | Jelaskan skala sensitifitas | Here we have a sensitivity scale. We can use this to see how sensitive people in the community think something is or isn't.  At one end, we have things that are not at all sensitive. Which means people will be very happy to discuss this.  At the other, we have topics that are VERY sensitive and that no-one likes or would be happy to talk about.  The darker the color, the more sensitive the topic. | Di sini kami memiliki skala sensitivitas. Kami bisa menggunakan ini untuk melihat seberapa bersedia masyarakat, membicarakan topik yang berbeda.  Di satu sisi, kami memiliki topik di mana masyarakat akan sangat senang berdiskusi dengan peneliti.  Di sisi lainnya, kami memiliki topik yang SANGAT sensitif dan masyarakat tidak senang membicarakan tentang itu dengan peneliti.  Semakin gelap warnanya, dan tinggi angkanya, semakin tidak bersedia membicarakan tentang topik itu. |
| agreescore_land | Your previous answers suggest that clearing forest in TNGL is….  ${senscore_land}  Do you feel this is accurate? | Jawaban anda sebelumnya menyatakan bahwa membuka hutan di TNGL adalah…   ${senscore_land}   Apakah Anda merasa ini akurat? | Show participant where they score on the sensitivity scale. | Tunjukan kepada peserta di mana nilai mereka pada skala sensitivitas. |
| why_land | Why? | Mengapa? |  |  |
| Section 6. Sensitive behaviour 2 – Hunting wildlife | | | | |
| hunting | Hunting wildlife | Berburu satwa liar |  |  |
| hunt_note | Hunting wildlife | Berburu satwa liar | These next questions will be about hunting wildlife. | Pertanyaan berikut adalah tentang berburu satwa liar. |
| comm_hunt | If we ask people in the community if they hunt wildlife in village land, how willing do you think people will be to talk to us honestly? | Jika kami bertanya kepada orang-orang di kelompok masyarakat apakah mereka berburu satwa liar di lahan desa, menurut Anda seberapa bersedia mereka untuk berkata jujur tentang itu ? |  |  |
| approval_hunt | If you personally hunted wildlife in village land, do you think your friends & family would... | Jika Anda sendiri berburu satwa liar di lahan desa, menurut Anda keluarga dan teman-teman Anda akan… |  |  |
| morals_hunt | To what extent do you agree with the following statement? | Sejauh mana Anda setuju dengan pernyataan ini? | *"It is good to hunt wildlife in village land"* | *"Adalah hal baik berburu satwa liar di lahan desa"* |
| sdb_hunt | To what extent do you agree with the following statement? | Sejauh mana Anda setuju dengan pernyataan ini? | *"If I hunted wildlife in village land, people in the community would think well of me"* | *"Jika saya berburu satwa liar di lahan desa, orang-orang di masyarakat akan memandang tinggi kepada saya"* |
| comfort_hunt | If you hunted wildlife on village land and a researcher asked you questions about that, how comfortable would you feel answering questions truthfully? | Jika Anda berburu satwa liar di lahan desa, dan peneliti menanyakan pertanyaan tentang hal tersebut, seberapa nyaman Anda akan menjawab pertanyaan tersebut secara jujur? |  |  |
| comwhy_hunt | Why would you be uncomfortable dicussing hunting wildlife on village land? | Kenapa Anda merasa tidak nyaman membicarakan tentang berburu satwa liar di lahan desa? |  |  |
| rules_hunt | Are there any rules about hunting wildlife in village land? | Apakah ada aturan tentang berburu satwa liar di lahan desa? |  |  |
| senscale_hunt | Explain the Sensitivity Scale card | Jelaskan skala sensitivitas | Here we have a sensitivity scale. We can use this to see how sensitive people in the community think something is or isn't.  At one end, we have things that are not at all sensitive. Which means people will be very happy to discuss this.  At the other, we have topics that are VERY sensitive and that no-one likes or would be happy to talk about.  The darker the color, the more sensitive the topic. | Disini kami memiliki skala sensitivitas. Kami bisa menggunakan ini untuk melihat seberapa bersedia masyarakat, membicarakan topik yang berbeda.  Di satu sisi, kami memiliki topik di mana masyarakat akan sangat senang berdiskusi dengan peneliti.  Di sisi lainnya, kami memiliki topik yang SANGAT sensitif dan masyarakat tidak senang membicarakan tentang itu dengan peneliti.  Semakin gelap warnanya, dan tinggi angkanya, semakin tidak bersedia membicarakan tentang topik itu. |
| agreescore_hunt | Your score for hunting wildlife on village land is   ${senscore_hunt}  Do you feel this is accurate? | Nilai Anda tentang berburu satwa liar di lahan desa adalah   ${senscore_hunt}  apakah Anda merasa ini akurat? | Show participant where they score on the sensitivity scale. | Tunjukan kepada peserta di mana nilai mereka pada skala sensitivitas. |
| why_hunt | Why? | Mengapa? |  |  |
| pa_hunt | If a researcher were to ask about hunting inside TNGL, would the level of sensitivity….. | Jika seorang peneliti menanyakan tentang berburu di dalam TNGL, seberapa tingkat sensitivitasnya | Select one |  |
| pahunt_why | Why? | Mengapa? |  |  |
| Section 7. Sensitive behaviour 3 – Logging | | | | |
| logging | Logging | Menebang kayu |  |  |
| pa_note | Logging | Menebang kayu | These next questions will be about entering TNGL to collect resources | Pertanyaan berikutnya adalah tentang masuk ke dalam TNGL untuk menebang kayu. |
| comm_log | If we ask people in the community if they enter TNGL to cut wood, how willing do you think people will be to talk to us honestly? | Jika kami bertanya kepada masyarakat jika mereka masuk ke dalam TNGL untuk menebang kayu, menurut Anda seberapa bersedia mereka untuk berkata jujur tentang itu ? |  |  |
| approval_log | If you personally entered TNGL to cut wood do you think your friends & family would... | Jika Anda sendiri masuk ke dalam TNGL untuk menebang kayu, menurut Anda, keluarga dan teman Anda akan…. |  |  |
| morals_log | To what extent do you agree with the following statement? | Sejauh mana Anda setuju dengan pernyataan ini? | *"It is good to cut wood in TNGL"* | *"Adalah hal yang baik menebang kayu di dalam TNGL"* |
| sdb_log | To what extent do you agree with the following statement? | Sejauh mana Anda setuju dengan pernyataan ini? | *"If I cut wood in the TNGL, people in the community would think well of me"* | *"Jika saya menebang kayu di dalam TNGL, orang di masyarakat ini akan berpikir baik tentang saya"* |
| comfort_log | If you cut wood in the TNGL and a researcher asked you about that, how happy would you be to answer questions honestly? | Jika Anda menebang pohon di TNGL, dan peneliti menanyakan pertanyaan tentang hal tersebut, seberapa nyaman Anda akan menjawab pertanyaan tersebut secara jujur? |  |  |
| comwhy_log | Why would you be uncomfortable cutting wood in the TNGL? | Mengapa Anda merasa tidak nyaman untuk membicarakan tentang menebang kayu di TNGL? |  |  |
| rules_log | Are there any rules about cutting wood in the TNGL? | Apakah ada aturan tentang menebang kayu di TNGL? |  |  |
| senscale_log | Explain the Sensitivity Scale card | Jelaskan skala sensitivitas | Here we have a sensitivity scale. We can use this to see how sensitive people in the community think something is or isn't.  At one end, we have things that are not at all sensitive. Which means people will be very happy to discuss this.  At the other, we have topics that are VERY sensitive and that no-one likes or would be happy to talk about.  The darker the color, the more sensitive the topic. | Di sini kami memiliki skala sensitivitas. Kami bisa menggunakan ini untuk melihat seberapa bersedia masyarakat, membicarakan topik yang berbeda.  Di satu sisi, kami memiliki topik di mana masyarakat akan sangat senang berdiskusi dengan peneliti.  Di sisi lainnya, kami memiliki topik yang SANGAT sensitif dan masyarakat tidak senang membicarakan tentang itu dengan peneliti.  Semakin gelap warnanya, dan tinggi angkanya, semakin tidak bersedia membicarakan tentang topik itu. |
| agreescore_log | Your score for cutting wood in the TNGL  ${senscore_log}.   Do you feel this is accurate? | Jawaban Anda sebelumnya menyatakan bahwa menebang kayu di TNGL adalah…   ${senscore_log}   Apakah Anda merasa ini akurat? | Show participant where they score on the sensitivity scale. | Tunjukan kepada peserta di mana nilai mereka pada skala sensitivitas. |
| why_log | Why? | Mengapa? |  |  |
| Section 8. Thank you & wrap-up | | | | |
| thank_you | The survey is now finished.   Thank you for participating. | Do you have any questions for me? | Survey ini sudah selesai. Terimakasih sudah berpartisipasi. | Apakah Anda memiliki pertanyaan untuk saya? |
| feedback | ${enumerator} have you any comments or feedback? | Record any comments or feedback If none, NA | Preferensi metode | Catat semua komentar dan pertanyaan, jika tidak "NA". |

## Survey Instrument – English and Kiswahili

Table S1c. Survey instrument used in Tanzania to collect data using ODK.

|  | **label::English** | **label::Kiswahili** | **hint::English** | **hint::Kiswahili** |
| --- | --- | --- | --- | --- |
| Section 1. Survey location | | | | |
| start_time |  |  |  |  |
| end_time |  |  |  |  |
| Date |  |  |  |  |
| enumerator | Who is conducting the interview? | Nani anafanya mahojianao? |  |  |
| region | Region | Mkoa |  |  |
| district | District | Wilaya |  |  |
| village | Name of the village | Jina la kijiji |  |  |
| pa_type | Nearest Protected Area | Hifadhi iliyokaribu hapa ni ipi | **Do not ask the respondent this** | **Usimwulize mhojiwa hili** |
| hi_present | Is Harriet present? | Harriet yupo? |  |  |
| Section 2. Consent | | | | |
| ethics_statement | Read the Consent Script to the participant | Soma fomu ya kuomba ridhaa ya mhojiwa | Hello. My name is ${enumerator} and I am helping Harriet Ibbett, who is from Bangor University in the UK to conduct research. Harriet’s research is all about understanding the best way to ask questions about natural resource use.  The survey has two parts. First of all, will ask your opinion about different types of natural resource use. The second part of the survey is to find out how you most prefer to answer questions about an activity that might be considered sensitive.   We are asking lots of people to complete this survey so that we can understand how people prefer to answer questions. Using your opinions and suggestions we will design a new survey, which will involve collecting information from local people about resource use around Protected Areas. The information you provide us is very important in making sure that we ask questions in the right way.   The questionnaire will take about 1hour 15 minutes to complete. Any information you provide will be anonymous, this means I will not record your name, or any information that can personally identify you or your household. Your answers will not be communicated to anyone in a form where your reply can be linked to you. I will record all your answers on this phone. All your answers will then be saved on a secure computer which can only be accessed by Harriet using a password.   At the end of the research, Harriet will write a report on her findings. This report will be used to help other researchers conduct research that better meets the needs of local people. Some results may also be published internationally so that other people in different countries can learn from our experience working with communities here.  Please note that we are independent, we are not related to the government or any NGOs and we have neutral views. We have permission of the Tanzanian government and the village chief to carry out this research. However, participation is voluntary. You do not have to participant and you stop participating at any time, without explanation. If you do I will discard your responses. If you feel uncomfortable answering some of the questions, you do not have to answer. If you would like to skip a question or a topic, please say.  This study has been reviewed by, and received ethics clearance through Bangor University. If you have any questions, please ask me and I will do my best to answer them.   If you remain unhappy or wish to make a formal complaint, I can give you the contact details of someone to discuss this with. | Habari, Jina langu ni ${enumerator} na ninamsaidia Harriet Ibbett anayetoka chuo kikuu cha Bangor Uingereza, kufanya utafiti. Utafiti wa Harriet unahusu kuelewa njia nzuri ya kuuliza maswali kuhusu utumiaji wa rasilimali asili.   Utafiti una sehemu mbili. Kwanza kabisa, tutakuuliza maoni yako kuhusu aina mbalimbali za matumizi ya rasilimali asili. Sehemu ya pili ya utafiti ni kuhusu jinsi unavyopenda kujibu maswali yanayoweza kuwa nyeti.  Tutawaomba watu wengi kujibu maswali ya utafiti huu ili tuweze kuelewa jinsi watu wanavyopenda kujibu maswali. Kwa kutumia maoni na mapendekezo yako tutaandaa utafiti mpya, ambao utahusisha ukusanyaji wa taarifa kutoka kwa watu, kuhusu utumiaji wa rasilimali karibu na maeneo yaliyohifadhiwa. Taarifa utakayotupatia ni ya muhimu sana katika kuhakikisha kwamba tutauliza maswali kwakutumia njia sahihi.  Mahojiano yanakadiriwa kuchukua takribani lisaa na robo. Taarifa yoyote utakayotupatia itakuwa ya siri, hii inamaana sitaandika jina lako au taarifa yoyote ambayo itaweza kukutambulisha binafsi au kaya yako. Majibu yako hayatatolewa kwa mtu yoyote kwa njia yoyote ambayo mtu anaweza kuhusianisha majibu yako na wewe.   Nitaandika majibu yako yote kwenye simu hii. Majibu yako yote yatahifadhiwa kwenye kompyuta salama ambayo inaweza kutumiwa na Harriet kwakutumia neno la siri. Mwisho wa utafiti Harriet ataandika ripoti ya majibu ya utafiti.   Taarifa hii itatumika kusaidia watafiti wengine kufanya tafiti zinazokidhi mahitaji ya jamii. Matokeo mengine yanaweza kuchapishwa kimataifa ili watu wengine katika mataifa mbalimbali waweze kujifunza kupitia uzoefu wetu wa kufanya kazi katika jamii za Tanzania.  Tafadhali kumbuka tunajitegemea na hatuhusiani na upande wowote, uwe wa Serikali au Mashirika yasiyokuwa ya serikali na tunamtazamo usiofungamana na upande wowote. Tuna kibali kutoka Serikali ya Tanzania na Serikali ya kijiji ya kufanya utafiti huu.   Japokuwa ushiriki ni wa hiari, na unaweza kujiondoa wakati wowote bila kutoa maelezo. Kama utajiondoa nitafuta majibu yako. Kama hautakuwa huru kujibu baadhi ya maswali unarususiwa kutokujibu. Kama utataka kuruka swali au mada tafadhali sema.  Utafiti huu umerejewa na kuruhusiwa na kamati ya maadili ya Chuo kikuu cha Bangor. Kama una swali lolote, tafadhali niulize na nitajitahidi niwezavyo kukujibu.  Kama utakuwa na wasiwasi au unataka kutoa malalamiko nitakupa mawasiliano ya mtu unayeweza kuzungumza naye. |
| consent | Did participant give their consent to participate? | Je, mhojiwa ametoa ridhaa yake ya ushiriki? |  |  |
| no_consent | Thank the participant and end the survey. | Mshukuru mshiriki na maliza mahojiano. |  |  |
| consent_gender | What was the gender of the participant? | Taja jinsia ya mshiriki |  |  |
| Section 3. Respondent demographics | | | | |
| gender | What gender is the participant? | Jinsia ya mshiriki |  |  |
| age | How old are you? | Una umri gani? | If unknown, ask them to estimate their age | Kama hawafahamu waombe wakadirie umri wao |
| ethn | What tribe are you? | Wewe ni kabila gani? |  |  |
| ethn_other | Please specify which tribe | Tafadhari ainisha kabila lako |  |  |
| language | What is the main language you speak? | Ipi ni lugha yako ya msingi unayozungumza? |  |  |
| language_other | Please specify which language | Tafadhali taja lugha hiyo |  |  |
| religious | Are you religious? | Una dini? |  |  |
| religion | What is your religion? | Dini yako ni ipi? |  |  |
| religion_other | Please specify which religion | Tafadhali taja |  |  |
| religion_den | What denomination are you? | Dhehebu lako ni lipi? |  |  |
| den_other | Please specify which dominion | Tafadhali taja dhehebu |  |  |
| religion_importance | How important is religion to you? | Dini ina umuhimu gani kwako? |  |  |
| yrs_ed | How many years of schooling do you have? | Umesoma kwa miaka mingapi? | Enter approximate number of years (max 13 years)  If none, enter 0  If unsure, write DK If diploma/degree etc - write this | Weka kadirio la miaka (isiyozidi miaka 13)    Kama hakuna weka 0  Kama hana uhakika, andika DK Kama ana diploma/shahada n.k. - andika |
| literacy | Can you read? | Unaweza kusoma? |  |  |
| literacy_ease | How do you find reading? | Ni rahisi kiasi gani kwako kusoma? | Read out options to respondent | Soma machaguo kwa mhojiwa |
| birth_month | Do you know the month in which you were born? | Je, unajua mwezi uliozaliwa? |  |  |
| month | Birth month | Mwezi uliozaliwa? |  |  |
| own_mobile | Do you personally own a mobile phone? | Unamiliki simu ya mkononi ambayo ni yako binafsi? | This means the individual. Not the household. | Hii inamaana mtu binafsi na sio kaya |
| know_pa | Do you know the name of the nearest Protected Area? | Unafahamu jina la hifadhi iliyoko karibu? |  |  |
| pa_name | Name of the nearest Protected Area | Jina la hifadhi |  |  |
| pa_other | If other, name of the Protected Area | Kama ni 'nyingine', taja jina la hifadhi |  |  |
| Section 4. Sensitive behaviour 1 – Livestock grazing | | | | |
| sens_note | Measuring sensitive behaviours | Kupima unyeti wa tabia | I will now ask you about a range of activities that you, members of your household, other family members, your friends or neighbours in the village might do.   For each activity, I will ask several questions. I don’t want to know whether you do these activities, but just about your opinions.  The first activity is grazing livestock. | Tungependa kukuuliza kuhusu shughuli mbalimbali ambazo wewe,, wana kaya wako na wanafamilia wengine,rafiki zako au jirani zako hapa kijijini wanaweza kufanya.   Kwa kila shughuli, tutakuuliza maswali machache.   Hatuhitaji kufahamu kama unafanya shughuli hizi lakini kupata maoni yako juu ya shughuli hizi.   Shughuli ya kwanza ni kuchunga mifugo |
| comm_lg | If we ask people in the community if they **graze livestock** inside the ${pa_type}, how willing do you think people will be to talk to us honestly about this? | Kama tukiuliza watu katika jamii kama wanachunga mifugo ndani ya ${pa_type}, unafikiri watakuwa na utayari kiasi gani kuongea na sisi kwa uwazi kuhusu hili? |  |  |
| approval_lg | If you personally grazed livestock in the ${pa_type}, do you think your friends & family would... | Kama wewe mwenyewe ulichunga mifugo ndani ya ${pa_type}, unafikiri ndugu na marafiki zako watafanyaje… |  |  |
| morals_lg | To what extent do you agree with the following statement? | Ni kwa kiasi gani unakubaliana na sentensi ifuatayo? | *"It is wrong to graze livestock in the ${pa_type}"* | *"Ni kosa kuchunga mifugo ndani ya ${pa_type}"* |
| sdb_lg | To what extent do you agree with the following statement? | Ni kwa kiasi gani unakubaliana na sentensi ifuatayo? | *"If I grazed livestock in the ${pa_type}, I would worry that people in the community would think less of me"* | *"Kama nilichunga mifugo ndani ya ${pa_type}, nitakuwa na wasiwasi wanajamii hawatanifikiria vizuri"* |
| rules_lg | Are there any rules about grazing livestock in the ${pa_type}? | Je, kuna sheria zozote zinazozuia kuchunga mifugo ndani ya ${pa_type}? |  |  |
| comfort_lg | If you grazed livestock inside the ${pa_type} and a researcher asked you questions about that, how comfortable would you feel to answer questions truthfully? | Kama wewe binafsi ulichunga mifugo ndani ya ${pa_type} na mtafiti akakuuliza maswali kuhusu hilo, utajisikia vizuri kiasi gani kujibu maswali kwa ukweli? |  |  |
| comwhy_lg | Why would you be uncomfortable dicussing livestock grazing in the ${pa_type}? | Kwa nini usiwe huru kuzungumza juu ya kuchunga mifugo ndani ya ${pa_type? |  |  |
| senscale_lg | Explain the Sensitivity Scale | Elezea kadi ya kipimo cha unyeti | Here we have a sensitivity scale. We can use this to see how sensitive people in the community think something is or isn't.  At one end, we have things that are not at all sensitive. Which means people will be very happy to discuss this.  At the other, we have topics that are VERY sensitive and that no-one likes or would be happy to talk about.  The darker the color, the more sensitive the topic. | Hapa tuna kipimo cha kiwango cha unyeti. Tunaweza kutumia hii kuona jinsi watu wanavyochukulia jambo fulani kuwa au kutokuwa nyeti.   Upande mmoja tunavyo vitu ambavyo sio nyeti kabisa, ina maanisha watu watafurahi sana kujadili juu ya jambo hilo.  Kwa upande mwingine tunayo mada ambayo ni nyeti sana na hakuna mtu angependa au angefurahi kuizungumzia.   Rangi inavyozidi au kuongezeka kukolea inaonyesha kuongezeka kwa unyeti wa mada. |
| agreescore_lg | Your previous answers suggest that grazing livestock in ${pa_type} is….  ${senscore_lg}  Do you feel this is accurate? | Alama zako kwa kuchunga mifugo ndani ya ${pa_type} ni   ${senscore_lg}   Unafikiri hii ni sahihi | Show participant where they score on the sensitivity scale. | Waonyeshe washiriki wanapata alama gani katika kipimo/uwiano wa unyeti. |
| why_lg | Why? | Kwa nini? |  |  |
| Section 5. Sensitive behaviour 2 – Bushmeat consumption | | | | |
| meat_note | Eating bushmeat | Kula nyamapori | These next questions will be about eating bushmeat. | Maswali yafuatayo yanahusu ulaji wa nyamapori. |
| qname_meat | Is there a local name used to describe bushmeat in this community? | Je, kuna jina linalotumika hapa kijijini kumaanisha nyamapori? |  |  |
| meat_name | What is this name? | Inaitwaje? |  |  |
| comm_meat | If we ask people in the community if they eat bushmeat, how willing do you think people will be to talk to us honestly? | Kama tukiuliza watu katika jamii kama wanakula nyamapori, unafikiri watakuwa na utayari kiasi gani kuongea na sisi kwa uwazi? |  |  |
| approval_meat | If you personally ate bushmeat, do you think your friends & family would... | Kama wewe mwenyewe umekula nyamapori, unafikiri rafiki na ndugu zako watafanyaje… |  |  |
| morals_meat | To what extent do you agree with the following statement? | Ni kwa kiwango gani unakubaliana na sentensi ifuatayo? | *"It is wrong to eat bushmeat"* | *"Ni kosa kula nyamapori"* |
| sdb_meat | To what extent do you agree with the following statement? | Ni kwa kiwango gani unakubaliana na sentensi ifuatayo? | *"If I ate bushmeat, I would worry that people in the community would think less of me"* | *"Kama nimekula nyamapori nitakuwa na wasiwasi wanajamii watanifikiria vibaya"* |
| rules_meat | Are there any rules about eating bushmeat? | Je, kuna sheria zozote zinazozuia kula wa nyamapori? |  |  |
| comfort_meat | If you ate bushmeat and a researcher asked you questions about that, how comfortable would you feel answering questions truthfully? | Kama wewe binafsi ulikula nyamapori na mtafiti akakuuliza maswali kuhusu hilo, utajisikia vizuri kiasi gani kujibu maswali kwa ukweli? |  |  |
| comfort_why_meat | Why would you be uncomfortable discussing eating bushmeat? | Kwanini usiwe huru kuzungumza/kujadili juu ya ulaji wa nyamapori? |  |  |
| senscale_meat | Explain the Sensitivity Scale card | Elezea kadi ya kipimo cha unyeti | Here we have a sensitivity scale. We can use this to see how sensitive people in the community think something is or isn't.  At one end, we have things that are not at all sensitive. Which means people will be very happy to discuss this.  At the other, we have topics that are VERY sensitive and that no-one likes or would be happy to talk about.  The darker the color, the more sensitive the topic. | Hapa tuna kipimo cha kiwango cha unyeti. Tunaweza kutumia hii kuona jinsi watu wanavyochukulia jambo fulani kuwa au kutokuwa nyeti.   Upande mmoja tunavyo vitu ambavyo sio nyeti kabisa, ina maanisha watu watafurahi sana kujadili juu ya jambo hilo.  Kwa upande mwingine tunayo mada ambayo ni nyeti sana na hakuna mtu angependa au angefurahi kuizungumzia.   Rangi inavyozidi au kuongezeka kukolea inaonyesha kuongezeka kwa unyeti wa mada. |
| agreescore_meat | Your score for eating bushmeat is   ${senscore_meat}  Do you feel this is accurate? | Alama zako kwa kula nyamapori ni  ${senscore_meat}   Unafikiri hii ni sahihi? | Show participant where they score on the sensitivity scale. | Waonyeshe washiriki wanapata alama gani katika kipimo/uwiano wa unyeti. |
| why_meat | Why? | Kwa nini? |  |  |
| pa_meat | If a researcher were to ask about eating bushmeat caught in ${pa_type}, would the level of sensitivity….. | Kama mtafiti angekuuliza kuhusu kula nyama pori iliyokamatwa ndani ${pa_type}, kiwango cha unyeti kinge... | Select one | Chagua moja hapa |
| pameat_why | Why? | Kwa nini ? |  |  |
| Section 6. Sensitive behaviour 3 – Hunting wildlife | | | | |
| hunt_note | Hunting wildlife | Kuwinda wanyamapori | These next questions will be about hunting wildlife. | Maswali yafuatayo yanahusu uwindaji wa wanyamapori. |
| comm_hunt | If we ask people in the community if they hunt wildlife in village land, how willing do you think people will be to talk to us honestly? | Kama tukiuliza watu katika jamii kama wanafanya uwindaji wa wanyama pori katika ardhi ya kijiji, unafikiri watakuwa na utayari kiasi gani kuongea na sisi kwa uwazi? |  |  |
| approval_hunt | If you personally hunted wildlife in village land, do you think your friends & family would... | Kama wewe mwenyewe umewinda wanyamapori katika ardhi ya kijiji, unafikiri rafiki na ndugu zako watafanyaje… |  |  |
| morals_hunt | To what extent do you agree with the following statement? | Ni kwa kiwango gani unakubaliana na sentensi ifuatayo? | *"It is wrong to hunt wildlife on village land"* | *"Ni kosa kuwinda wanyamapori katika ardhi ya kijiji"* |
| sdb_hunt | To what extent do you agree with the following statement? | Ni kwa kiwango gani unakubaliana na sentensi ifuatayo? | *"If I hunted wildlife on village land, I would worry that people in the community would think less of me"* | *"Kama nikiwinda wanyamapori katika ardhi ya kijiji nitakuwa na wasiwasi wanajamii watanifikiria vibaya"* |
| rules_hunt | Are there any rules about hunting wildlife in village land? | Je, kuna sheria zozote zinazozuia uwindaji wa wanyamapori katika ardhi ya kijiji? |  |  |
| comfort_hunt | If you hunted wildlife on village land and a researcher asked you questions about that, how comfortable would you feel answering questions truthfully? | Kama wewe binafsi uliwinda wanyamapori katika ardhi ya kijiji na mtafiti akakuuliza maswali kuhusu hilo, utajisikia vizuri kiasi gani kujibu maswali kwa ukweli? |  |  |
| comfort_why_hunt | Why would you be uncomfortable discussing hunting wildlife on village land? | Kwanini usiwe huru kuzungumza juu ya uwindaji wanyamapori katika ardhi ya kijiji? |  |  |
| senscale_hunt | Explain the Sensitivity Scale card | Elezea kadi ya kipimo cha unyeti | Here we have a sensitivity scale. We can use this to see how sensitive people in the community think something is or isn't.  At one end, we have things that are not at all sensitive. Which means people will be very happy to discuss this.  At the other, we have topics that are VERY sensitive and that no-one likes or would be happy to talk about.  The darker the color, the more sensitive the topic. | Hapa tuna kipimo cha kiwango cha unyeti. Tunaweza kutumia hii kuona jinsi watu wanavyochukulia jambo fulani kuwa au kutokuwa nyeti.   Upande mmoja tunavyo vitu ambavyo sio nyeti kabisa, ina maanisha watu watafurahi sana kujadili juu ya jambo hilo.  Kwa upande mwingine tunayo mada ambayo ni nyeti sana na hakuna mtu angependa au angefurahi kuizungumzia.   Rangi inavyozidi au kuongezeka kukolea inaonyesha kuongezeka kwa unyeti wa mada. |
| agreescore_hunt | Your score for hunting wildlife on village land is   ${senscore_hunt}  Do you feel this is accurate? | Alama zako kwa kuwinda ni wanyamapori katika ardhi ya kijiji  ${senscore_hunt}   Unafikiri hii ni sahihi? | Show participant where they score on the sensitivity scale. | Waonyeshe washiriki wanapata alama gani katika kipimo/uwiano wa unyeti. |
| why_hunt | Why? | Kwa nini? |  |  |
| pa_hunt | If a researcher were to ask about hunting inside the ${pa_type}, would the level of sensitivity….. | Kama mtafiti angekuuliza kuhusu kuwinda wanyamapori ndani ya ${pa_type}, kiwango cha unyeti kinge... | Select one | Chagua moja hapa |
| pahunt_why | Why? | Kwa nini? |  |  |
| Section 7. Sensitive behaviour 3 – Entering PA to collect resources | | | | |
| pa_note | Entering ${pa_type} to collect resources | Kuingia ndani ya ${pa_type} kuokota rasilimali | These next questions will be about entering ${pa_type} to collect resources | Maswali yafuatayo yatahusu kuingia ndani ya ${pa_type} kuokota rasilimali |
| comm_pa | If we ask people in the community if they enter ${pa_type} to collect resources, how willing do you think people will be to talk to us honestly? | Kama tukiuliza watu katika jamii kama wanaingia ndani ya ${pa_type} kuokota rasilimali, unafikiri watakuwa na utayari kiasi gani kuongea na sisi kwa uwazi? |  |  |
| approval_pa | If you personally entered ${pa_type} to collect resources do you think your friends & family would... | Kama wewe mwenyewe umeingia ndani ya ${pa_type} kuokota rasilimali, unafikiri rafiki na ndugu zako watafanyaje… |  |  |
| morals_pa | To what extent do you agree with the following statement? | Ni kwa kiwango gani unakubaliana na sentensi ifuatayo? | *"It is wrong to enter ${pa_type} to collect resources "* | *"Ni kosa kuingia ndani ya ${pa_type} kuokota rasilimali"* |
| sdb_pa | To what extent do you agree with the following statement? | Ni kwa kiwango gani unakubaliana na sentensi ifuatayo? | *"If I entered ${pa_type} to collect resources, I would worry that people in the community would think less of me"* | *"Kama nikiingia ndani ya ${pa_type} kuokota rasilimali, nitakuwa na wasiwasi wanajamii watanifikiria vibaya"* |
| rules_pa | Are there any rules about entering ${pa_type} to collect resources? | Je, kuna sheria zozote zinazozuia kuingia ndani ya ${pa_type} kuokota rasilimali? |  |  |
| comfort_pa | If you entered ${pa_type} to collect resources and a researcher asked you about that, how happy would you be to answer questions honestly? | Kama wewe binafsi uliingia ndani ya ${pa_type} kuokota rasilimali na mtafiti akakuuliza maswali kuhusu hilo, utajisikia vizuri kiasi gani kujibu maswali kwa ukweli? |  |  |
| comwhy_pa | Why would you be uncomfortable discussing entering ${pa_type} to collect resources? | Kwanini usiwe huru kuzungumza juu ya kuingia ndani ya ${pa_type} kuokota rasilimali? |  |  |
| senscale_pa | Explain the Sensitivity Scale card | Elezea kadi ya kipimo cha unyeti | Here we have a sensitivity scale. We can use this to see how sensitive people in the community think something is or isn't.  At one end, we have things that are not at all sensitive. Which means people will be very happy to discuss this.  At the other, we have topics that are VERY sensitive and that no-one likes or would be happy to talk about.  The darker the color, the more sensitive the topic. | Hapa tuna kipimo cha kiwango cha unyeti. Tunaweza kutumia hii kuona jinsi watu wanavyochukulia jambo fulani kuwa au kutokuwa nyeti.   Upande mmoja tunavyo vitu ambavyo sio nyeti kabisa, ina maanisha watu watafurahi sana kujadili juu ya jambo hilo.  Kwa upande mwingine tunayo mada ambayo ni nyeti sana na hakuna mtu angependa au angefurahi kuizungumzia.   Rangi inavyozidi au kuongezeka kukolea inaonyesha kuongezeka kwa unyeti wa mada. |
| agreescore_pa | Your score for entering ${pa_type} to collect resources is   ${senscore_pa}.   Do you feel this is accurate? | Alama zako kwa kuingia ndani ya ${pa_type} kuokota rasilimali ni   ${senscore_pa}.   Unafikiri hii ni sahihi? | Show participant where they score on the sensitivity scale. | Waonyeshe washiriki wanapata alama gani katika kipimo/uwiano wa unyeti. |
| why_pa | Why? | Kwa nini? |  |  |
| Section 8. Thank you & wrap-up | | | | |
| thank_you | The survey is now finished.   Thank you for participating. | Mwisho wa mahojiano  Asante kwa kushiriki | Do you have any questions for me? | Una maswali yoyote ya kuniuliza? |
| feedback | ${enumerator} have you any comments or feedback? | ${enumerator}, una maoni yoyote au mrejesho? | Record any comments or feedback  If none, NA | Andika maoni yoyote au mrejesho |

# Appendix 2. Data analysis

## Descriptive statistics for response items measured with psychometric scale

Table S2a. Descriptive statistics for psychometric scale items included in the exploratory factor analysis. n indicates number of rows where responses were obtained for all five scale items, across all behaviours.

| **Response item** | **n** | **Median** | **Mean** | **SD** | **SE** | **Skew** | **Kurtosis** |
| --- | --- | --- | --- | --- | --- | --- | --- |
| *Indonesia* | | | | | | | |
| Injunctive norm | 849 | 4 | 3.38 | 1.02 | 0.04 | -0.46 | -0.91 |
| Moral attitude towards behaviour | 849 | 4 | 3.30 | 1.02 | 0.04 | -0.42 | -1.07 |
| Social desirability of behaviour | 849 | 4 | 3.53 | 0.90 | 0.03 | -0.98 | -0.04 |
| Personal comfort discussing behaviour | 849 | 3 | 3.07 | 1.02 | 0.03 | -0.07 | -1.47 |
| Community willingness to discuss behaviour | 849 | 3 | 2.93 | 1.00 | 0.03 | 0.25 | -1.40 |
| *Tanzania* | | | | | | | |
| Injunctive norm | 1025 | 4 | 3.88 | 0.61 | 0.02 | -1.53 | 4.45 |
| Moral attitude towards behaviour | 1025 | 4 | 4.05 | 0.67 | 0.02 | -1.80 | 6.95 |
| Social desirability of behaviour | 1025 | 4 | 3.89 | 0.61 | 0.02 | -1.85 | 5.94 |
| Personal comfort discussing behaviour | 1025 | 4 | 3.62 | 0.79 | 0.02 | -1.22 | 0.79 |
| Community willingness to discuss behaviour | 1025 | 4 | 3.51 | 1.01 | 0.03 | -1.10 | 0.21 |

Table S2b. Knowledge of rules regarding the behaviours asked about in the questionnaire.

| **Indonesia** | | | | **Tanzania** | | | | |
| --- | --- | --- | --- | --- | --- | --- | --- | --- |
| Do you know if there are any rules about… | Yes | No | Don’t know | Do you know if there are any rules about… | Yes | No | Don’t know | No response |
| Hunting on village land (n=281) | 33% | 65% | 2% | Hunting on village land (n=263) | 88% | 4% | 9% | - |
| Clearing land in the NP (n=279) | 91% | 5% | 4% | Eating bushmeat (n=250) | 91% | 2% | 7% | 1% |
| Logging in the NP (n=289) | 97% | 2% | 1% | Grazing livestock in the PA (n=248) | 95% | 1% | 3% | - |
|  |  |  |  | Entering the PA to collect resources (n=264) | 93% | 1% | 5% | 1% |

Table S2c. Changes in perceived sensitivity of hunting, when hunting conducted in protected areas

| **If a researcher were to ask about hunting inside the protected area, would the level of sensitivity…** | **Indonesia**  **(n=301)** | **Tanzania**  **(n=278)** |
| --- | --- | --- |
| Increase a lot | 10% | 20% |
| Increase a little | 62% | 10% |
| Stay the same | 19% | 49% |
| Decrease a little | 1% | 1% |
| Decrease a lot | 0 | 0 |
| Don’t know | 8% | 20% |

Figure S2a. Percentage of responses in each category for each item in the psychometric scale used to measure topic sensitivity. Responses to the right of the centre line (grey shaded area) indicate the proportion of responses that suggest the behaviour is sensitive, while responses on the left suggest low sensitivity. Note that the response are presented in reverse for the last two statements in Tanzania (i.e. strongly agree appears to the right of 0, rather than the left).

## Exploratory Factor Analysis of psychometric scale data

### Indonesia

Figure S2b. Scatter plots, histograms and Pearson correlation coefficients for each item in the Indonesia dataset (n=849).


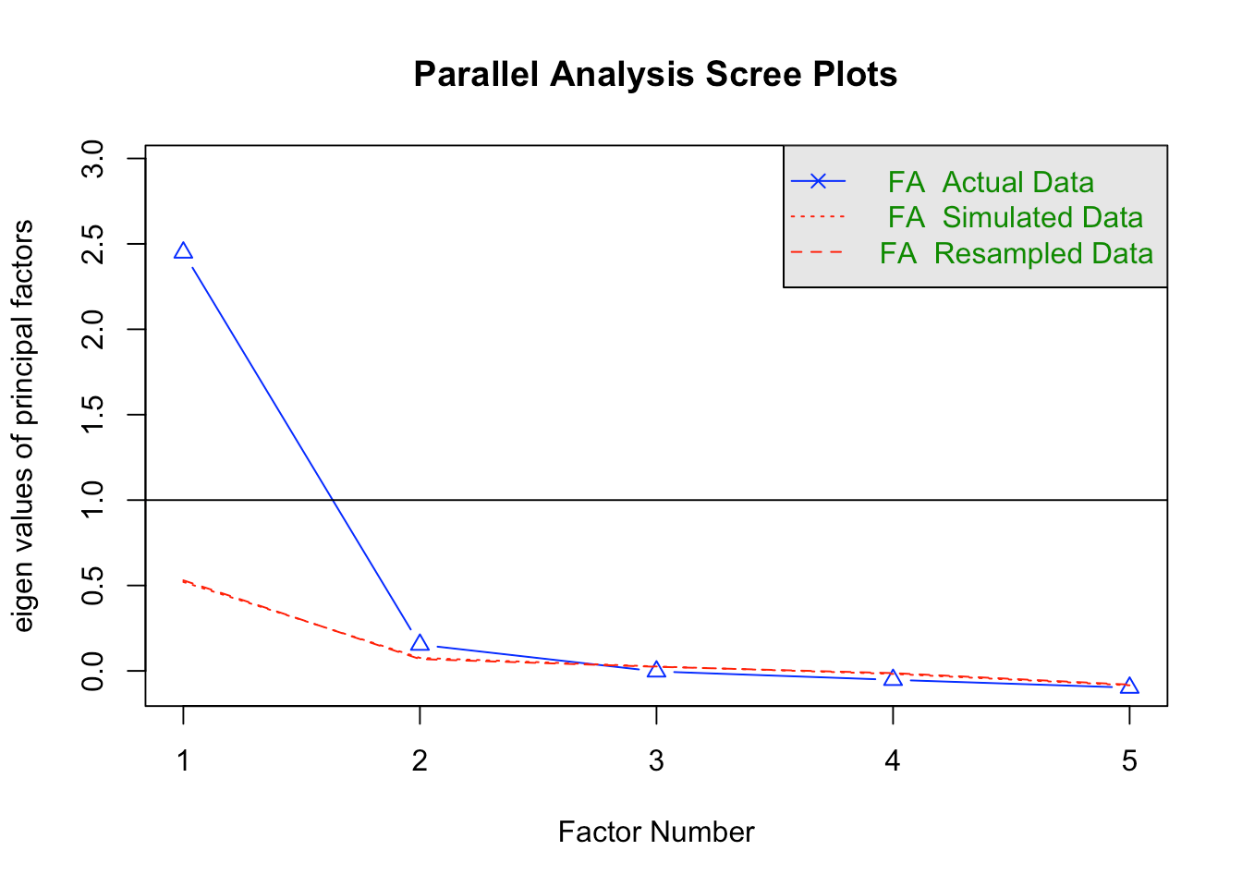


Figure S2c. Scree plot of eigen values for Indonesian dataset (n= 849).

Table S2d. Results from 1-factor and 2-factor Exploratory Factor Analyses run on Indonesia dataset.

| **Indonesia** | | | | |
| --- | --- | --- | --- | --- |
|  | **1-factor EFA loadings** |  | **2-factor EFA loadings** | |
| *Item* | *Factor1* |  | *Factor1* | *Factor2* |
| Injunctive norm | 0.68 |  | 0.56 | - |
| Moral attitude | 0.77 |  | 0.87 | - |
| Social desirability | 0.62 |  | 0.61 | - |
| Personal comfort | 0.69 |  | - | 0.56 |
| Community willingness to discuss | 0.61 |  | - | 0.78 |
| Sum of Squared loadings | - |  | 1.59 | 1.07 |
| Proportional Variance | - |  | 0.32 | 0.21 |
| Cumulative Variance | - |  | 0.32 | 0.53 |
| Degrees of freedom & fit | 5, 0.07 |  | 1 |  |
| n | 849 |  | 849 |  |
| Likelihood Chi-Sq | 61.84 with prob < 0.001 |  | 0.84 with prob <0.36 | |
| RMSEA Index | 0.116 90%CI (0.091, 0.142) |  | 0.000 (90%CI 0, 0.08) | |
| Tucker Lewis Index | 0.91 |  | 1.001 |  |
| SRMR | 0.05 |  | 0 | |
| BIC | 28.12 |  | -5.91 |  |

Cut-offs for good model fit: chi-square test of exact fit, Root Mean Square Error of Approximation (RMSEA ≤ 0.06), Tucker Lewis Index (TLI ≥ 0.95), Standardized Root Mean Square Residual (SRMR ≤ 0.08) (Boatang et al. 2018), Bayesian Information Criteria (BIC < as possible)

### Tanzania

Figure S2e. Scatter plots, histograms and Pearson correlation coefficient for each item in the Tanzania dataset (n=1025).


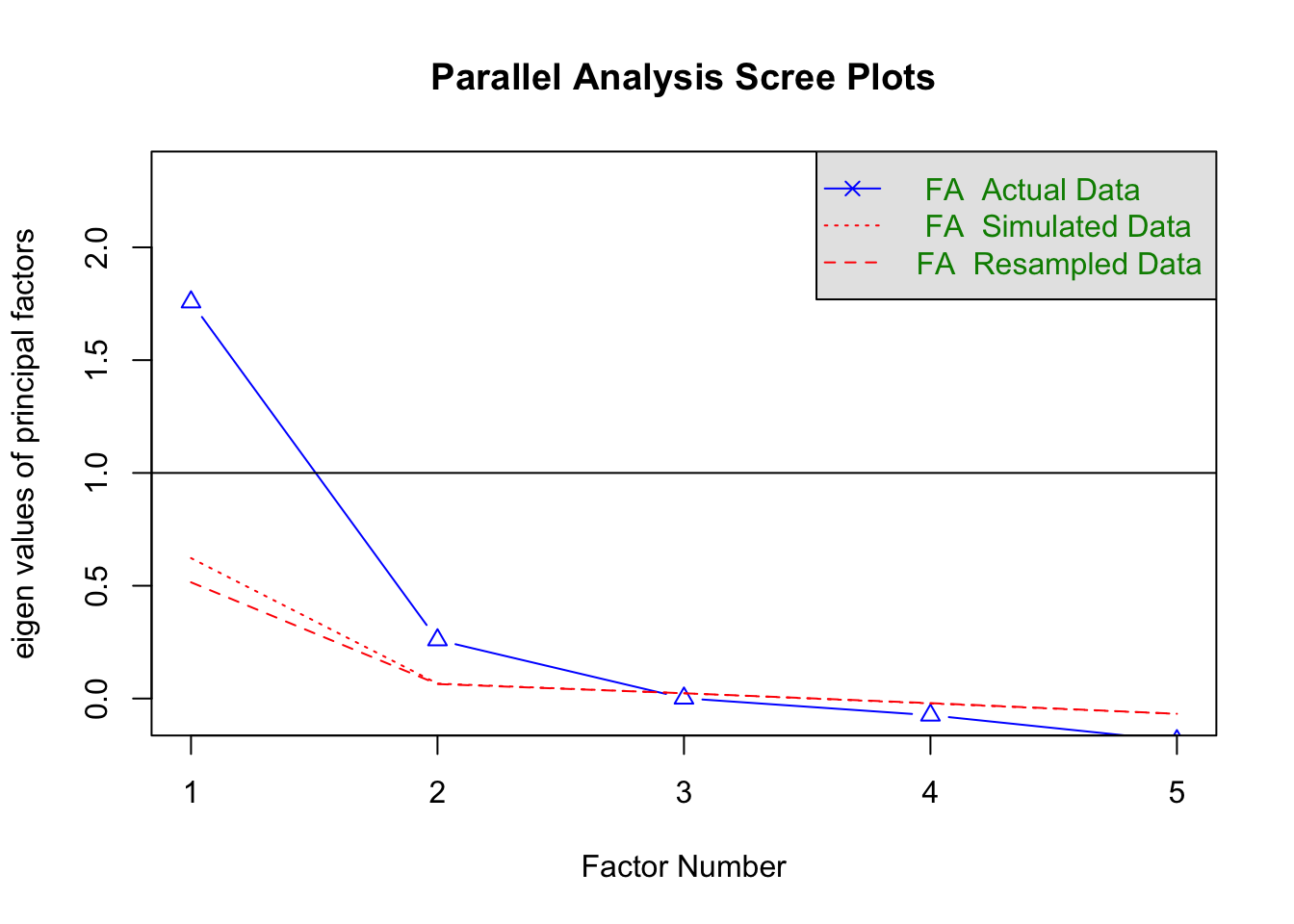


Figure S2f. Scree plot of eigen values for Tanzania (n=1025)

Table S2e. Results from 1-factor and 2-factor Exploratory Factor Analyses run on Tanzania dataset.

| **Tanzania** | | | | |
| --- | --- | --- | --- | --- |
|  | **1-factor EFA loadings** |  | **2-factor EFA loadings** | |
| *Item* | *Factor1* |  | *Factor1* | *Factor2* |
| Injunctive norm | 0.60 |  | 0.401 | - |
| Moral attitude | 0.66 |  | 0.727 | - |
| Social desirability | 0.77 |  | 0.846 | - |
| Personal comfort | 0.41 |  | - | 0.663 |
| Community willingness to discuss | 0.32 |  | - | 0.538 |
| Sum of Squared loadings | - |  | 1.406 | 0.804 |
| Proportional Variance | - |  | 0.281 | 0.161 |
| Cumulative Variance | - |  | 0.281 | 0.442 |
| Degrees of freedom | 5 |  | 1 |  |
| n | 1025 |  | 1025 |  |
| Likelihood Chi-Sq | 151.8 with prob <0.001 |  | 5.69 with prob <0.017 | |
| RMSEA Index | 0.169 90%CI (0.147, 0.193) |  | 0.068 (90%CI 0.023, 0.126) | |
| Tucker Lewis Index | 0.697 |  | 0.952 |  |
| SRMR | 0.09 |  | 0.01 | |
| BIC | 117.14 |  | -1.25 |  |

Cut-offs for good model fit: chi-square test of exact fit, Root Mean Square Error of Approximation (RMSEA ≤ 0.06), Tucker Lewis Index (TLI ≥ 0.95), Standardized Root Mean Square Residual (SRMR ≤ 0.08) (Boatang et al. 2018), Bayesian Information Criteria (BIC < as possible)

## Sensitivity Index

### Creation of a composite Sensitivity Index

The composite index for sensitivity was created by obtaining factor-scores from the 2-factor EFA model. We extracted factor-scores using the ‘tenBerge’ method in ‘pysch’ package (Revelle, 2021), which produces factor scores that preserve correlation between factors (i.e. when using an oblique factor rotation). These were transformed from z-scores, to a scale ranging from 0 to 1. For each row of data, we multiplied the transformed factor-score for each factor, by the proportion ratio each factor represented, to create a weighted factor-score. The proportion ratio for each factor was calculated as:

$$Proportion ratio= \frac{Proportion of variance represented by the factor}{Total variance explained by all factors}$$

The composite Sensitivity Index was calculated by summing these transformed weighted factor-scores:

$$Sensitivity Index = {(F}_{1}* R_{1}) + {(F}_{2}* R_{2})$$

Where:

$F$ = Transformed factor-score identified for each factor, for each row of data

$R$ = Proportion ratio for the factor (i.e. the proportion of variance explained by the factor, transformed into a ratio)

_1, 2, …_ = The id of the factor identified in the model (e.g. 1 = Factor1, 2 = Factor2)

### Beta regression models

Table S2f. Explanation of the response and predictor variables tested in country-specific Beta regression models with mixed-effects

| **Variables** | | **Description *(Data type: Levels)*** |
| --- | --- | --- |
| Response variable | | |
|  | Sensitivity Index | Composite index created from summing weighted factor-scores  *(Continuous: ranging from 0 to 1)* |
| Predictor variables *(Effect type)* | | |
|  | ID *(Random effect) ^a^* | Unique ID code assigned to each respondent *(Continuous)* |
|  | Age | Age of respondents in years *(Continuous)* |
|  | Gender | Gender of the respondent *(Categorical: Male / Female)* |
|  | Education | Number of years schooling the respondent completed *(Continuous)* |
|  | Behaviour | Behaviour the respondent was asked about  *(Categorical: Indonesia – hunting in village land, logging in the National Park, Clearing land in the National Park;*  *Tanzania – hunting in village land, eating bushmeat, grazing livestock in the nearest protected area, entering the protected area)* |
|  | Knowledge of rules | Whether the respondent knew if there were any rules associated with the behaviour (categorical: Yes / No) |
|  | Type of Protected Area | The type of protected area the respondent lived nearest to *(Categorical: Indonesia – National Park, Protected Forest, Other; Tanzania – National Park, Game Reserve, Other)* |
| Interactions |  |  |
|  | Behaviour X Protected area type |  |
|  | Behaviour X Knowledge of rules |  |

^a^ Included as a random effect to control for respondents answering multiple questions per behaviour

### Can simplistic analyses on smaller samples provide similar results to the Sensitivity Index?

Psychometric scales require large samples, which are not always possible to obtain during the preliminary design stages of research. Recognising this as a limitation of our Sensitivity Index, we wanted to test whether a simple analysis, conducted using a more feasible sample size (e.g. 40 individuals) would produce similar results to those obtained with weighted-factor-scores.

To do so, we randomly extracted responses from 40 individuals from each country dataset, and summarised the mean raw response for each behaviour. We repeated this process 10 times, each time extracting a different subset of 40 individuals. Results are plotted below (Fig. S2g) alongside the Sensitivity Index calculated using weighted-factor scores for the whole sample. Although they are portrayed using different y-axis, broadly speaking, the patterns of sensitivity are the same, suggesting crude analysis using raw data, can provide an indicator of sensitivity equal to that obtained using more complex analyses.

Figure S2g. Raw response scores show the mean score obtained for each behaviour, in each country (Indonesia, left; Tanzania, right) from a subset of 10 randomly generated samples of 40 respondents. Each coloured dot represents the mean score of the five items used to measure the sensitivity of each behaviour. Weighted-factor-scores, shows the mean Sensitivity Index calculated for the whole dataset using weighted-factor-scores. Dark grey dots represent the mean Sensitivity Index, other coloured dots show the distribution of the data.

## Free-listing analysis and results

### Calculating item salience

The following formula can be used to calculate salience of each item:

$$Item salience =\frac{1+{length}_{i}-{rank}_{i}}{{length}_{i}}$$

Where length indicates the number of items listed in focus group $i$, and rank reflects the position of the item in the list of focus group $i$. Smiths salience, which reflects the overall importance of an item in the sample, is calculated by summing the salience scores for each item listed, and dividing it by the total number of lists ($n$):

$$Smiths Salience = \frac{\Sigma{item salience}_{i}}{n}$$

### Results from the first free-listing exercise (Reasons why people go to Protected Areas)

Table S2g. Reasons for going to protected areas listed by group participants in Indonesia (10 groups, 60 participants) and Tanzania (8 groups, 66 participants), ordered by Smith’s Salience, with number groups mentioning an item (n). Items mentioned did not differ by gender, table combines results from both genders. Items marked by an * are those that are freely allowed within protected areas, ^p^ indicate activities that are allowed in some protected areas with a permit, all other activities are prohibited.

| **Indonesia** (26 items listed) | Salience | n | **Tanzania** (20 items listed) | | Salience | n |
| --- | --- | --- | --- | --- | --- | --- |
| Farming | 0.63 | 7 | Grazing livestock | | 0.49 | 6 |
| Collecting firewood | 0.50 | 8 | Collecting timber to build houses | | 0.49 | 6 |
| Collecting flowers | 0.28 | 4 | Fishing | | 0.48 | 5 |
| Working as a tour guide * | 0.24 | 4 | Collecting honey | | 0.41 | 5 |
| Hunting wild pig | 0.24 | 3 | Collecting water ^p^ | | 0.40 | 5 |
| Collecting birds | 0.22 | 3 | Hunting wildlife | | 0.37 | 4 |
| Collecting rattan | 0.20 | 4 | Farming | | 0.28 | 4 |
| Clearing land | 0.20 | 1 | Collecting firewood | | 0.27 | 4 |
| Collecting wood for building (logging) ^p^ | 0.18 | 2 | Collecting grass for roofs | | 0.25 | 5 |
| Collect damar resin | 0.13 | 1 | Mining | | 0.24 | 3 |
| Collecting herbs/plants for medicine | 0.10 | 3 | Charcoal making | | 0.20 | 3 |
| Hunting monkey | 0.10 | 2 | Collecting medicine | | 0.18 | 4 |
| Tapping palm trees | 0.10 | 1 | Employment * | | 0.13 | 1 |
| Tapping rubber trees | 0.09 | 1 | Collecting wood for sculptures | | 0.12 | 2 |
| Collecting grass | 0.08 | 1 | Visiting relatives ^p^ | | 0.07 | 1 |
| Grazing cattle | 0.08 | 1 | Worship ^p^ | | 0.06 | 3 |
| Grazing livestock & collecting grass | 0.07 | 1 | Collecting natural fibres to make rope | | 0.06 | 1 |
| Hunting wildlife | 0.06 | 2 | To sell products ^p^ | | 0.05 | 1 |
| Fishing | 0.05 | 2 | To see the airplane | | 0.02 | 1 |
| Collecting wild plants | 0.05 | 2 | To run away from home | | 0.01 | 1 |
| Fixing the pipelines from spring * | 0.05 | 1 |  |  |  |  |
| Collecting bamboo | 0.04 | 1 |  |  |  |  |
| Collecting cinnamon seeds | 0.03 | 1 |  |  |  |  |
| Collecting gaharu (agarwood) | 0.03 | 1 |  |  |  |  |
| Collecting rattan fruit | 0.03 | 1 |  |  |  |  |
| Checking the border of the National Park * | 0.02 | 1 |  |  |  |  |

### Qualitative notes from second free-listing exercise (Challenges of living alongside PAs)

#### Indonesia

In Indonesia, several groups reported that village populations were growing, and that protected area boundaries limited their ability to expand farms. One group explained that farms were often handed down through generations, and as a result many preceded the protected area. However, when the protected area was established, the boundary markers were incorrectly laid, absorbing their farmlands. As a result, some participants reported being unable to clear or cultivate their lands. In another group, participants explained that although most people in their community were farmers, not everyone owned land. The presence of the protected area prevented those without land from obtaining any. Although some households had permission to grow crops on state land near protected areas, lack of land tenure meant there was no long-term security in doing so.

The other main challenge listed by participants in Indonesia was damage to property and crops from wildlife. One group reported predation of livestock by tiger, and that species such as sun bear *(Helarctos malayanus),* elephant and orangutan visited the village. They said they received no compensation for livestock losses to tiger but did for crops (such as durian) damaged by orangutan. Another group reported issues with wild pig, and primates such as macaque *(Macaca sp.)*, langur *(Presbytis sp.)* and orangutan destroying crops. They reported using snares and electric fences to protect farms but said these were also dangerous as they posed a risk to people. Three groups reported no challenges to living alongside protected areas.

Focus group participants specifically requested to highlight that there were many benefits obtained from living alongside protected forest areas. These included clean water and air, protection from extreme weather events such as flooding, as well the provision of jobs through tourism.

#### Tanzania

In Tanzania, challenges of living alongside wildlife, particularly large, dangerous species were highly salient, particularly around Game Reserves. Elephant, bush pig *(Potamochoerus larvatus)*, ground hornbill *(Bucorvus leadbeateri)* and primates were reported to damage crops, while livestock was lost to carnivores such as lion, leopard *(Panthera pardus pardus)*, spotted hyena *(Crocuta crocuta)* and jackal *(Canis sp.)*. Participants also highlighted the risk to people from wildlife. One group relayed how a villager walking on the road at night had been attacked by hyena, and that a man had been killed and eaten by lion after falling asleep in the bush. In other groups, participants reported villagers being killed by elephant while protecting crops and defending food stores.

Issues associated with the location of Ruaha National Park boundary were listed by participants living near the park. Several groups reported that the National Park boundary had been moved several times without, they felt, adequate consultation or compensation, resulting in communities reporting that they had lost access to water sources, ancestral lands (including burial grounds and worship sites) and agricultural and grazing lands. Participants explained that movement of the boundary had led to feelings of anxiety, distrust towards the National Park and a sense of powerlessness, with participants describing that they were unclear where ‘true’ boundaries now lay. One group was hesitant to complete the construction of a school, because they were afraid boundaries would move again. Changes to the National Park boundary was also reported as problematic because it reduced the land available for pastoralists to graze livestock, which in turn, exacerbated conflict within communities, particularly between pastoralists and agriculturalists who were left competing for land on which to graze livestock and grow crops.

Legislation relating to the establishment of protected areas in Tanzania varies with protected area type. According to Article 6 of the National Park Act (1959), the President can declare any area of land to be a National Park and can alter the boundaries of a National Park to include any area of land not already included. In such cases all rights, titles, interests, franchises, claims, privileges, exemptions or immunities of any person in respect of any land within the area ceases, and are forever extinguished. Any person who has any rights to land is entitled to compensation, providing claims are submitted within ten weeks of the date of the proclamation. The southern extent of Ruaha National Park was formerly Usangu Game Reserve, gazetted as such in 1998. According to the Wildlife Conservation Act (2009) the President may also declare any area of Tanzania to be a Game Reserve, and, under Article 14.1, should consult with relevant local authorities first. The designation of wildlife corridors, dispersal areas, buffer zones and migratory routes is decentralised to the Minister, who may, in consultation with relevant local authorities order such gazettements. In 2008, Usangu Game Reserve was incorporated into Ruaha National Park (Wildlife Conservation Act 1974 – Wildlife Conservation (Usangu Game Reserve Declaration) Order – Government Notice 436A of 1998). The change in protected area status followed the eviction of hundreds of cattle herders and their animals from the reserve (Walsh 2012). According to government, under the Wildlife Conservation Act (2009), the Usangu Game Reserve land was already reserved land, and not village land as suggested by participants in our focus groups. Contemporary complaints concerning the National Park boundary reported by our participants, and others (see Zia et al. 2011) likely reflect the turbulent historic context of protected area establishment in the Usangu area.

The relationship between communities and protected area law enforcement was raised in one capacity or another in all groups. Some groups living near Game Reserves reported that rangers, tasked with enforcing protected area rules, assisted communities when they had problems with wildlife. However, other groups living around the National Park reported poor relations with rangers, reporting stories of misconduct, corruption, and violence. One group felt rangers often went beyond their authority. They described a recent incident whereby rangers had searched homes in the middle of the night, shot bullets into the air, and beaten those suspected of wrong-doing. Another group reported that rules were enforced unequally. For example, they explained that it was legal to fish, however, if you were unable to prove where your catch came from, rangers may arrest you for fishing in the protected area. Another group said that if rangers searched your home and found beef meat, they would claim it was bushmeat and impart sanctions. In several groups, participants reported that villagers had been arrested on their farms (particularly during rainy season), simply because their farms were located close to the protected area. Issues related to power and law enforcement also came up within these groups. For example, one group located far from the main village (and closer to the National Park), reported that they felt powerless against rangers because they were so far from the protection of their community leader, and that they were disadvantaged as rangers only spoke Kiswahili (whereas some villagers only spoke their ethnic language); if a villager was unable to speak Kiswahili, they were unable to explain or defend themselves. In several groups, participants said that threatening to report community members to rangers for rule-breaking (e.g., for hunting wildlife) was used to leverage power over others.

Other challenges listed included that protected areas restricted communities’ access to essential resources (e.g., water and firewood), restricted opportunities for development (e.g., authorities would not allow the construction of power lines to the village through the protected area), with tsetse flies being reported as a specific issue around Game Reserves. Participants reported that they received few benefits from protected areas; unlike the ‘safari circuit’ in the north of the country, there was little tourist infrastructure in the landscape.
